# miR-1/133a Clusters Cooperatively Specify the Cardiomyogenic Lineage by Adjustment of Myocardin Levels during Embryonic Heart Development

**DOI:** 10.1371/journal.pgen.1003793

**Published:** 2013-09-19

**Authors:** Katharina Wystub, Johannes Besser, Angela Bachmann, Thomas Boettger, Thomas Braun

**Affiliations:** Max-Planck-Institut für Herz- und Lungenforschung, Department of Cardiac Development and Remodelling, Bad Nauheim, Germany; Medizinische Hochschule Hannover, Germany

## Abstract

miRNAs are small RNAs directing many developmental processes by posttranscriptional regulation of protein-coding genes. We uncovered a new role for miR-1-1/133a-2 and miR-1-2/133a-1 clusters in the specification of embryonic cardiomyocytes allowing transition from an immature state characterized by expression of smooth muscle (SM) genes to a more mature fetal phenotype. Concomitant knockout of miR-1-1/133a-2 and miR-1-2/133a-1 released suppression of the transcriptional co-activator myocardin, a major regulator of SM gene expression, but not of its binding partner SRF. Overexpression of myocardin in the embryonic heart essentially recapitulated the miR-1/133a mutant phenotype at the molecular level, arresting embryonic cardiomyocytes in an immature state. Interestingly, the majority of postulated miR-1/133a targets was not altered in double mutant mice, indicating that the ability of miR-1/133a to suppress target molecules strongly depends on the cellular context. Finally, we show that myocardin positively regulates expression of miR-1/133a, thus constituting a negative feedback loop that is essential for early cardiac development.

## Author Summary

miRNAs are small non-coding RNAs involved in posttranscriptional regulation of protein-coding genes. In the mammalian genome, two distinct gene clusters code for miR-1 and miR-133a. Primary sequences of mature miR-1 or miR-133a are identical and both gene clusters show similar expression in the heart and skeletal muscle. We have generated compound mutant mice of both miR-1/133a gene clusters resulting in early arrest of heart development while single cluster mutants showed normal morphology but reacted differently to pressure overload. Compound mutant cardiomyocytes were characterized by an immature, mixed smooth muscle-heart muscle phenotype, indicating that miR1-/133a are responsible for specification of the cardiomyogenic lineage. Our search for miR1-/133a targets identified myocardin, which was strongly up-regulated in mutant hearts, while several other putative miR-1/133a targets that have been described before were not altered, indicating that miR-1/133a target control strongly depends on the cellular context. Overexpression of myocardin in embryonic hearts recapitulated major aspects of the miR-1/133a mutant phenotype, suggesting that loss of myocardin suppression is the primary reason for incorrect heart muscle specification in the mutants. In addition, we found that myocardin overexpression stimulated expression of miR-1/133a, which argues for a negative feedback loop required for adjustment of myocardin concentrations in the heart.

## Introduction

The mammalian heart is the earliest functional organ of the embryo. Ventricular contractions continuously provide blood supply to the developing embryo despite major morphological and functional reorganization of the heart during embryogenesis [1]. Coordination of this complex task is accomplished by a tightly regulated concert of cellular and molecular interactions. An example is the maturation of cardiomyocytes in the embryonic heart, which initially express smooth muscle genes but lose this expression when heart development progresses [2]–[4]. So far, relatively little is known about regulatory mechanisms controlling the transition between immature and mature cardiomyocytes that express smooth muscle genes only under stress conditions or during dedifferentiation [5].

miRNAs have been recognized in recent years as part of the regulatory networks that govern developmental or physiological processes. Heart specific deletion of the enzyme Dicer, essential for generation of miRNAs, and of individual miRNA genes revealed critical functions of miRNA-mediated regulation at various stages of cardiac development (for review see [6], [7]). Several miRNAs, which play a role during heart development, are specifically expressed in the heart or skeletal muscle such as miR-1/133a miRNAs or the so-called myomiRs located in introns of muscle-specific genes. The function of intronic myomiRs has been addressed in a number of elegant papers suggesting functions mainly under cardiac stress and in disease conditions [8], [9] while the exact role of miRNAs miR-1 and miR-133a is less clear, in part due to putative compensatory actions of these highly similar miRNAs. However, diseases of the heart also go along with changes of miR-1/133a expression similar to intronic myomirs, although it is often not clear whether such changes are due to an increase of non-cardiomyocytes in diseased hearts [10].

In the mammalian genome two distinct gene clusters located on two different chromosomes encode miR-1 and miR-133a: the miR-1-1/133a-2 and the miR-1-2/133a-1 cluster. Primary sequences of mature miR-1 or miR-133a are identical and both gene clusters show similar expression patterns suggesting that these miRNAs serve at least partially overlapping functions. A third miRNA cluster on mouse chromosome 1, related to miR-1/miR133a, encodes for miR-206 and miR-133b. In contrast to the miR-1/miR133a cluster, miR-206 and miR-133b are expressed mainly in somites during skeletal muscle development [11] and later become confined to slow skeletal muscle fibers. All three loci produce bicistronic transcripts containing one miRNA from the miR-1/206 family and one from the miR-133 family essentially forming functional units [12] that are under the transcriptional control of heart and muscle specific regulatory programs [13], [14]. Potential overlapping functions of miR-133a-1 and miR-133a-2 have been investigated by deletion of miR-133a coding regions without impairing miR-1 expression. Interestingly, concomitant deletion of both miR-133a genes causes a fetal heart phenotype of variable penetrance with ventricular septum defects (VSD) suggesting that miR-133a does not play a major role in early embryonic development. Surviving miR-133a mutants showed dilated cardiomyopathy with increased proliferation of cardiomyocytes and increased smooth muscle cell gene expression. The phenotype of miR-133a double mutants has been primarily ascribed to the loss of miR-133a-mediated repression of cyclinD2 and SRF [15]. In contrast to the analysis of miR-133a-1 and miR-133a-2 double mutants, only single miR-1-2 mutants have been analyzed. Deletion of miR-1-2 has been reported to cause VSDs leading to reduced survival of mutant mice [16]. In addition, defects in cell cycle regulation and cardiac conduction have been attributed to the up-regulation of the putative miR-1 target molecules Hand2 and Irx5, as well as of the Kcnd2 potassium channel [16]. miR-1 overexpression has been shown to decrease the pool of proliferating ventricular cardiomyocytes [14] and to attenuate cardiomyocyte hypertrophy by targeting molecules involved in calcium signaling [17].

Here, we analyzed the function of the miR-1-1/133a-2 and miR-1-2/133a-1 clusters for early cardiac development by targeted gene inactivation. Deletion of single miR-1/133a clusters did not lead to major developmental defects and did not impair viability of adult mice while deletion of both miR-1/133a gene clusters caused early embryonic lethality due to severe heart malformations. Transcriptional profiling of miR-1/133a double mutant hearts revealed an up-regulation of genes characteristic for immature cardiomyocytes. Transgenic overexpression of the newly discovered miR-1 target myocardin recapitulated major aspects of the miR-1/133a phenotype. We concluded that miR-1 and miR-133a control the faithful expression of genes in a functionally redundant manner by adjustment of myocardin levels to allow specification of early cardiomyocytes with hybrid expression of cardiomyocyte and smooth muscle specific markers to more differentiated fetal cardiomyocytes.

## Results

### Mice with targeted inactivation of individual *miR-1/133a* cluster are viable and fertile and show no gross morphological aberrations

**Figure 1:**
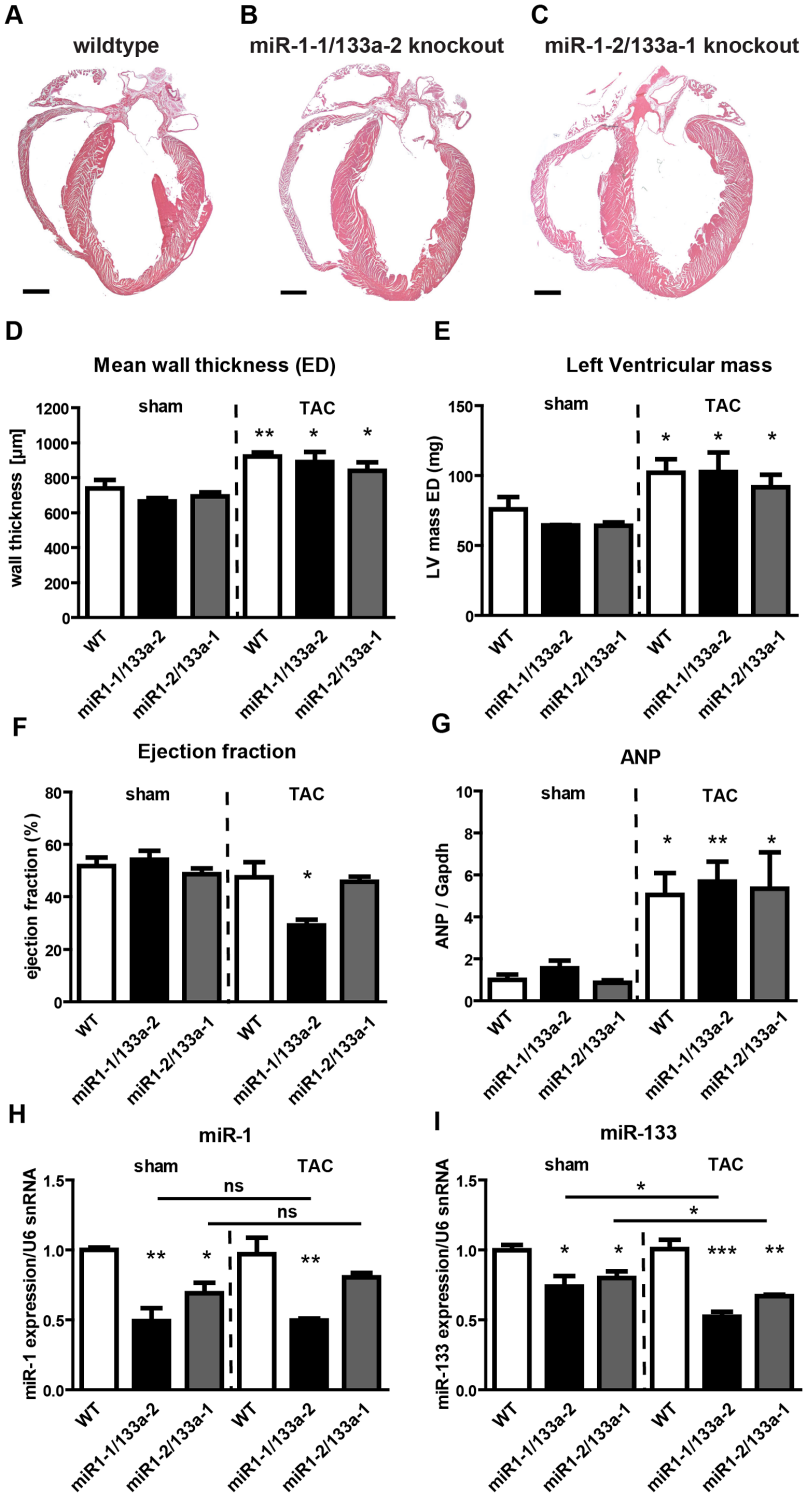
Deletion of single *miR-1/133a* clusters does not cause gross morphological alterations in the heart but results in decreased ejection fraction in *miR-1-1/133a-2* mutants after TAC. (A–C) No morphological abnormalities are discernable on frontal sections through hearts of *miR-1-1/133a-2* and *miR-1-2/133a-2* homozygous mutants. (D, E) Transverse aortic constriction led to an increase in wall thickness (D) and left ventricular mass (E) in comparison to sham-operated mice wildtype or *miR-1/133a* single *knock-out* mice as measured by MRI. (F) *miR-1-1/133a-2* but not *miR-1-2/133a-2* homozygous mutants showed a reduction in ejection fraction compared to wild type mice. (G) TAC-induced pressure overload resulted in a comparable increase in ANP levels in single cluster mutants and wild type controls. (H, I) qRT-PCR analysis (Taqman) of miR-1 and miR-133a expression in different single cluster mutant strains after TAC. No significant increase of miR-1 expression in *miR-1-1/133a-2* and *miR-1-2/133a-1* mutants after TAC compared to sham-operated mice while expression levels of miR-133a dropped slightly after TAC in both single cluster mutants.

The two miRNA *miR-1/133a* clusters constitute functional units at mouse chromosome 2 and chromosome 18 as both miRNAs are expressed in the heart and skeletal muscle as bi-cistronic messages. Mature miR-1-1/miR-1-2 and miR-133a-2/miR-133a-1 differ from each other indicating different target genes. In contrast, mature miR-1-1 is identical to miR-1-2 and miR-133a-2 is identical to miR-133a-1, suggesting potentially overlapping functions. To resolve the biological function of *miR-1/133a* clusters *in vivo*, we generated *knock-out* mice for each individual cluster (Suppl. Fig. S1). Mice mutant for single *miR-1/133a* cluster were born at the expected Mendelian ratio and were viable with survival rates identical to WT littermates (Suppl. Table S1). We did not observe gross developmental defects or histological aberrations in heart and muscle. (Fig. 1A–C, Suppl. Fig. S2A, B). These findings are in stark contrast to a previous study reporting ventricular septum defects, cardiomyocyte hyperplasia and increased nuclear division of cardiomyocytes after inactivation of *miR-1-2* [16]. Furthermore, ECG analysis did not reveal signs of arrhythmias or alterations of the QRS-complex in either single cluster *knock-out* strains (data not shown). Again, these findings differ from observations reported by Zhao et al. describing changes in the heart rate, shortened PR-interval, and a bundle-branch block in *miR-1-2* mutants [16]. At present, the reasons for these differences are unclear although several explanations seem possible (see discussion).

**Figure 2:**
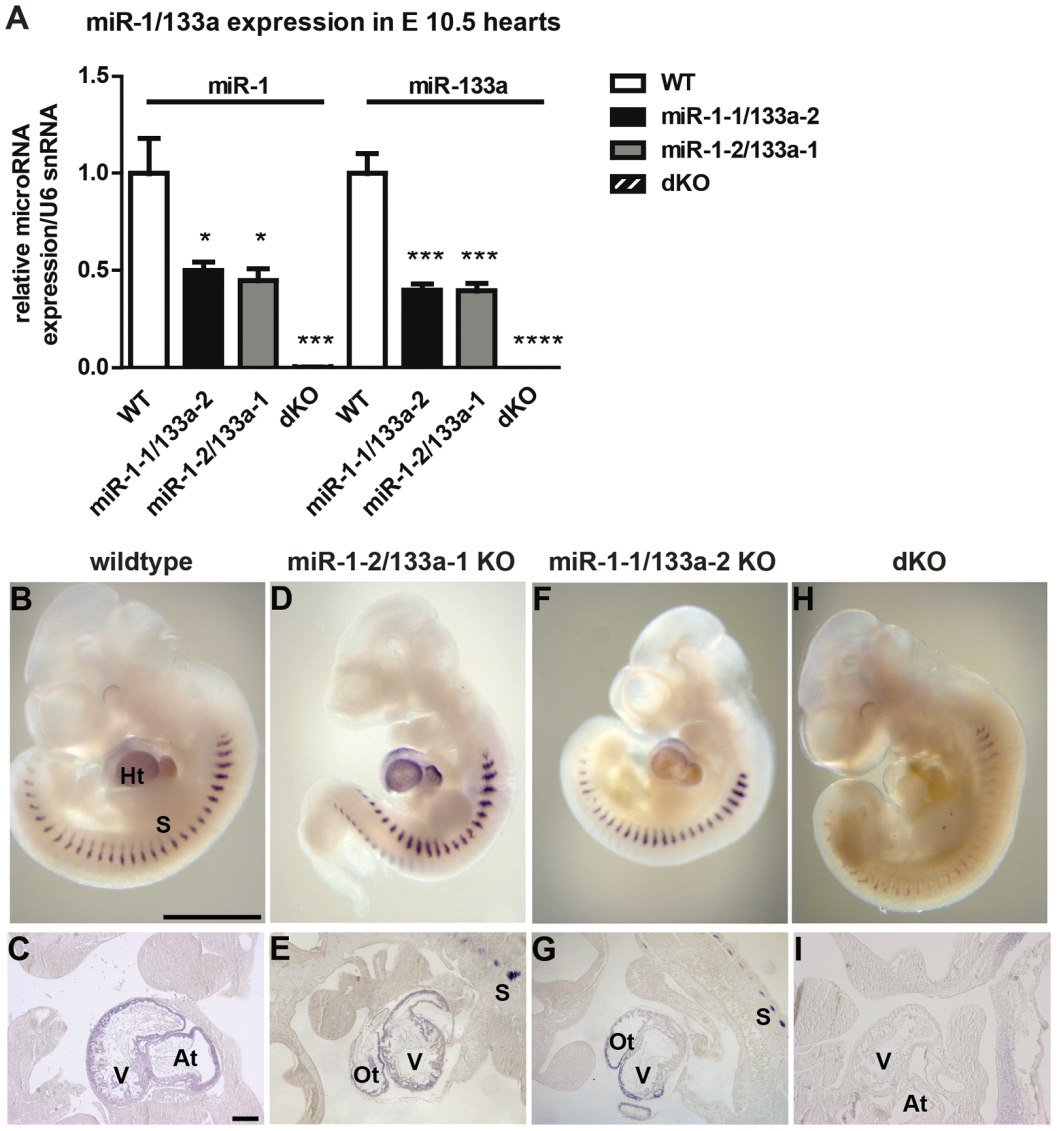
*miR-1/133a* clusters contribute equally to miR-1/133a expression in the developing heart. (A) Quantitative RT-PCR analysis of ca. 50% reduced expression of miR-1 and miR-133a expression in single cluster *knock-out* embryonic hearts and complete loss in *miR-1/133* dKO embryonic hearts at E10.5 using Taqman probes. (B–H) *Whole mount in situ* (WISH) expression analysis of miR-1 in mutant and WT embryos using LNA oligonucleotides. (C–I) Cryosections of WISH embryos. (D, E) Deletion of the *miR-1-2/133a-1* cluster uncovers expression of miR-1-1. (F, G) Deletion of the *miR-1-1/133a-2* cluster uncovers expression of miR-1-2. (H, I) Deletion of both clusters confirms specificity of miR-1 signals in the heart. Residual staining in somites might be due to cross hybridization with miR-206, which is not expressed in the heart. Scale bar in (B) corresponds 1000 µm in B, D, F, H, scale bar in (C) corresponds to 200 µm in C, E, G, I. at: atrium, ht: heart, ot: outflow tract, s: somites, v. ventricle.

Next, we analyzed heart functions of single *miR-1/133a* cluster mutants by cardiac magnetic resonance imaging (MRI) both under baseline condition and after pressure overload induced by transverse aortic constriction (TAC). No significant changes in mean wall thickness, left ventricular mass, and ejection fraction (EF) were detected in single cluster mutants compared to wildtype controls under baseline conditions (Fig. 1D–F). After TAC, we detected an increase in mean wall thickness and left ventricular mass and ANP expression, which did not differ between *miR-1-1/133a-2*, *miR-1-2/133a-1* mutants and wildtype controls (Fig. 1D–G). Interestingly, we found a significant reduction of the ejection fraction in *miR-1-1/133a-2* knockout mice while *miR-1-2/133a-1* mutants maintained the same normal ejection fraction as wildtype controls indicating that individual *miR-1/133a* clusters contribute differently to cardiac remodeling in response to pressure overload (Fig. 1F). Analysis of miR-1 and miR-133a concentrations in individual cluster mutants revealed no significant change of miR-1 expression after TAC compared to sham-operated mice (Fig. 1H). Expression levels of miR-133a dropped slightly after TAC in both single cluster mutants suggesting that the respective remaining *miR-1/133a* gene cluster possesses only a limited ability to react to the loss of individual alleles by increased expression both under baseline and pathological conditions (Fig. 1I). Similarly, we did not detect a compensatory increase of miR-1 and miR-133a expression in embryonic hearts of single miRNA cluster *knock-out* mice at E10.5 (Fig. 2A). However, it is difficult to exclude that the lack of a single cluster already leads to increased compensatory activity of the other cluster, thereby concealing the original contribution of individual clusters in wildtype animals.

### miRNAs miR-1/133a are essential for early cardiac development

**Figure 3:**
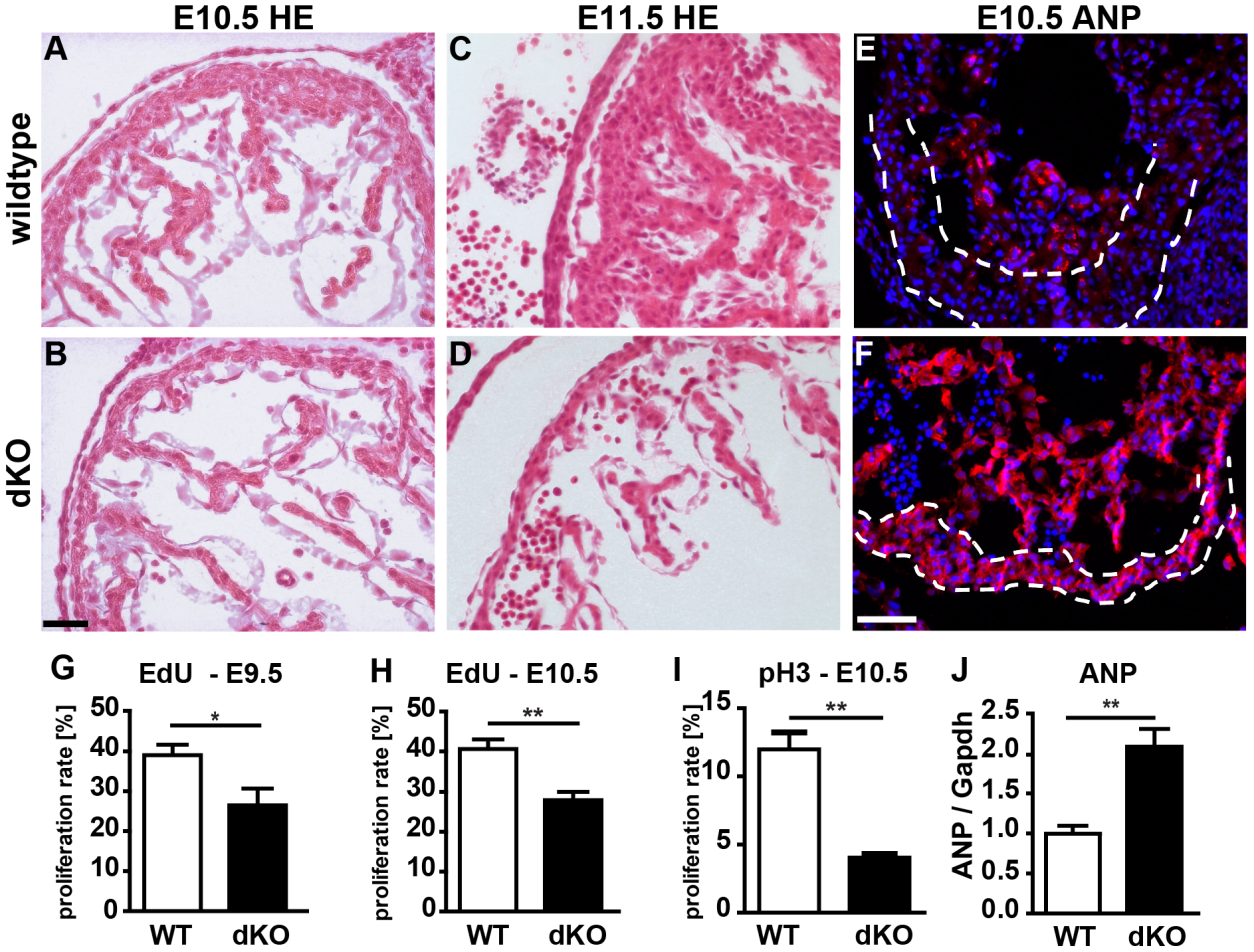
Loss of *miR-1/133a* leads to aberrant heart development and causes embryonic lethality. (A, B) Morphological analysis of heart development at E10.5 and (C, D) E11.5 using H&E stained sections. Arrest of heart development at E10.5 and reduced diameter of the compact layer of the ventricular wall in *miR-1/133a* dKO embryos are clearly visible. (E, F) Immunofluorescence analysis of ANP up-regulation in the compact layer of *miR-1/133a* dKO embryonic hearts. (F) The thinned compact layer of *miR-1/133a* dKO hearts expresses high levels of ANP. (G–I) Quantitative evaluation of reduced proliferation of cardiomocytes in *miR-1/133a* dKO hearts at E9.5 and E10.5 by EdU incorporation (G, H) and pH3 staining (I). (J) Quantitative RT-PCR analysis (Taqman) of increased expression of ANP in miR-1/133a dKO hearts. The scale bar in B corresponds to 50 µm in A–D. The scale bar in F corresponds to 100 µm in E–F.

Next, we generated mice that lack both clusters and hence completely fail to express miR-1 and miR-133a. Crosses of double heterozygous or compound heterozygous/homozygous animals did not yield viable double homozygous mutant animals (dKO). Analysis of different developmental stages revealed that dKO animals did not survive embryonic stage E11.5. A massive impairment of embryonic blood circulation and heart beating was visible in dKO embryos at E11.5 (Suppl. Fig. S3) and no living dKO embryos were found after E11.5 (Suppl. Table S2). Expression analysis at E10.5 confirmed a complete loss of miR-1 and miR-133a expression in dKO embryos (Fig. 2A,H, I). The pattern of miR-1 expression was not altered in single cluster mutants as visualized by *whole mount in situ* hybridization using LNA-probes (Fig. 2B–G) again indicating that miR-1-1 and miR-1-2, respectively miR-133a-1 and miR-133a-2 might substitute for each other. Since the *miR-1-2/133a-1* cluster is located in an intron of the *mib1* (*mindbomb*) gene, a component of the Notch and Wnt pathways, we analyzed expression of mib1 in wt and dKO embryos at E10.5 by qRT-PCR. No significant differences in expression levels or splicing patterns were observed in dKO versus wt embryos (Suppl. Fig. S4). Morphological analysis of E10.5 and E11.5 dKO embryos revealed severe developmental defects in heart development leading to thinning of the ventricular wall of the developing heart (Fig. 3A–F). Of note, we observed a striking reduction of the number of cells within the compact layer of the heart at E10.5 while the trabecular part was less affected. No further growth of compact and trabecular layers was apparent at E11.5 most probably due to a global arrest of heart development in dKO embryos (Fig. 3D). A more detailed analysis of the proliferation rate revealed a reduction of EdU incorporating myocardial cells in the compact cell layer of the developing myocardium at E9.5 and at E10.5 (Suppl. Fig. S5A–D, Fig. 3G, H). Similarly, we found a major reduction of pH3-positive cardiomyocytes identified by expression of myosin heavy chain in E10.5 dKO mutant embryos (Suppl. Fig. S5E, F, Fig. 3I). We also detected a strong up-regulation of the cardiac stress marker atrial natriuretic peptide (ANP) in the compact layer of dKO mutant embryos at E10.5 by immunofluorescence staining (Fig. 3F) and by RT-PCR (Fig. 3J) but no evidence for increased apoptotic cell death as measured by staining for activated caspase 3 (data not shown). In wild type hearts, expression of ANP at this developmental stage was mostly confined to the trabecular layer (Fig. 3E) further supporting the view that the compact layer was more severely affected than the trabecular layer by the loss of miR-1/133a although some morphological abnormalities in the trabecular layer were present as well.

**Figure 4:**
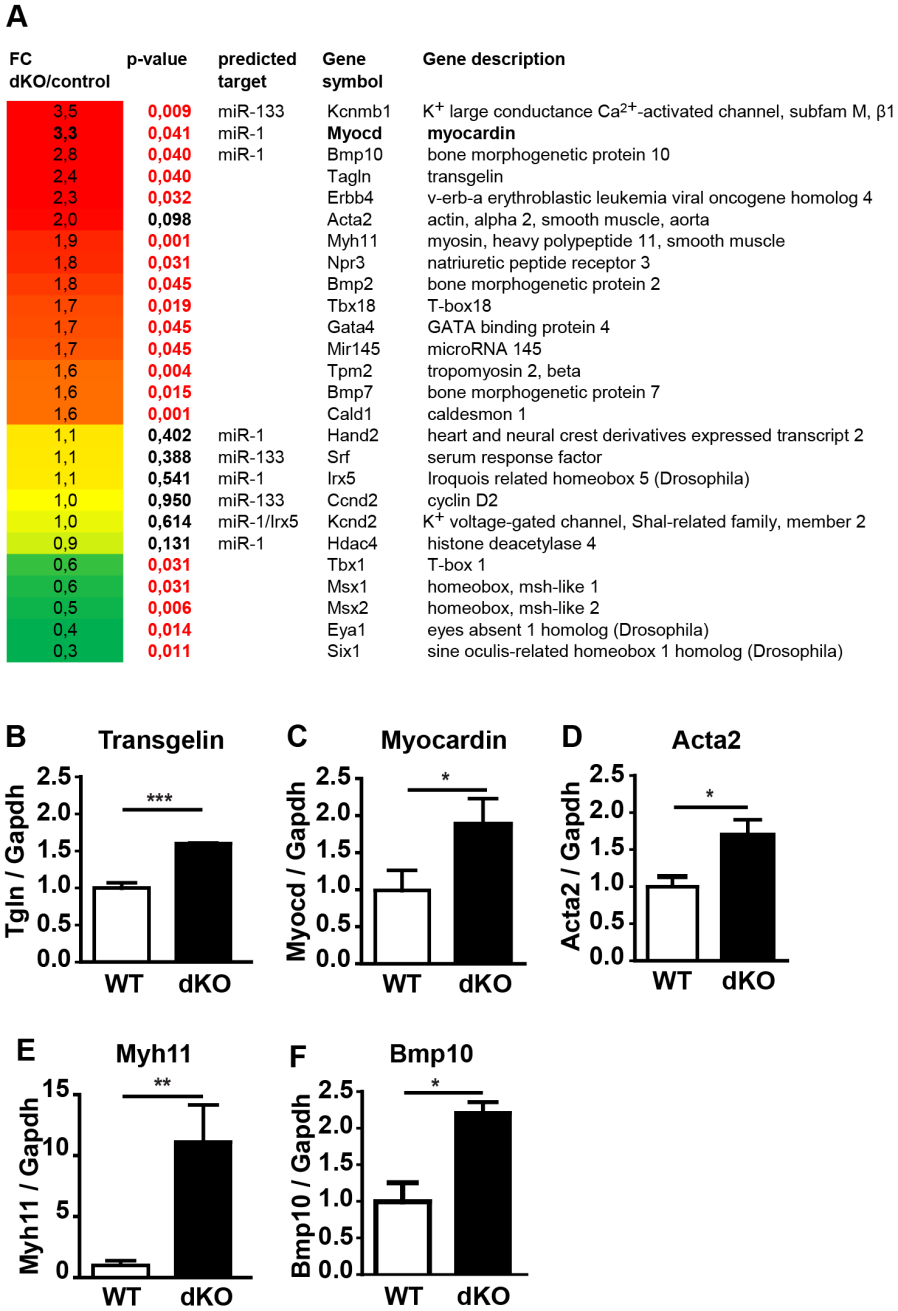
Deletion of *miR-1/133a* clusters induces up-regulation of smooth muscle-specific genes leading to multiple transcriptional changes in embryonic hearts. (A) DNA microarray-based transcriptional analysis of *miR-1/133a* dKO mutant hearts at E10.5. Genes associated with heart development showing significant (red: p-values) expression changes are shown (up-regulated genes: red, down-regulated genes: green). Putative miR-1/133a target genes are indicated. (B–F) qRT-PCR analysis (Taqman) of increased expression of ANP, myocardin, smooth muscle actin, transgelin, myh11 and BMP-10 in *miR-1/133a* dKO hearts.

To get insights into the molecular processes that are affected by loss of miR-1/133a we performed a comparative RNA expression analysis of the developing heart using RNA isolated from E10.5 hearts (n = 4, dKO; n = 5, controls). Data obtained by Affymetrix Genechip analysis were validated by specific quantitative RT-PCR Taqman assays using independent samples (n = 3/3). Unbiased gene ontology enrichment analysis using genes that were at least 1.5-fold up-regulated in *miR-1/133a* dKO compared to control hearts at E10.5 revealed that terms subsumed in the category “cell differentiation” showed the most significant enrichment. Other categories at the same level within the gene ontology hierarchy displayed significant lower p-values (Suppl. Fig. S6). Importantly, we identified a strong enrichment of terms associated with cardiomyocyte and smooth muscle cell differentiation suggesting that miR1/133a repress genes involved smooth/striated muscle differentiation. *Myocardin* and *Kcnmb1* were the strongest up-regulated genes (Fig. 4A, C) together with a consistent up-regulation of several smooth muscle markers such as *transgelin* (Fig. 4B), *smooth muscle actin (Acta2)* (Fig. 4D), *myh11* (Fig. 4E), *caldesmon* and *miR-145* (Fig. 4A). Increased expression of smooth muscle actin in dKO compared to wt cardiomyocytes was confirmed by immunofluorescence analysis of E10.5 hearts (Suppl. Fig. S5G, H). Analysis of transcriptional changes in mutant hearts also revealed increased expression of trabecular markers like *BMP-10* [18] and *Erbb4* [19] (Fig. 4A, F). Moreover, we observed changes in several genes involved in heart development, which probably reflects secondary events due to the global arrest of heart development (Fig. 4A). Specifically, we detected increased expression of *BMP-2*, *Gata4*, *Tbx18* and *BMP-7* and consistent down-regulation of *Msx1* and *Msx2*, which are involved in epithelial to mesenchymal transition and cardiac valve formation [20]. In addition, we saw a down-regulation of the *Tbx1*-*Six1*-*Eya1* axis essential for morphogenesis of the outflow tract [21] (Fig. 4A). The molecular data reflected morphological alterations in dKO hearts at E10.5 and suggested that repression of molecules characteristic for immature cardiomyocytes might be an important function of miR-1/133 in the developing heart.

### Myocardin and Kcnmb1 are primary targets of miR-1 and miR-133a

**Figure 5:**
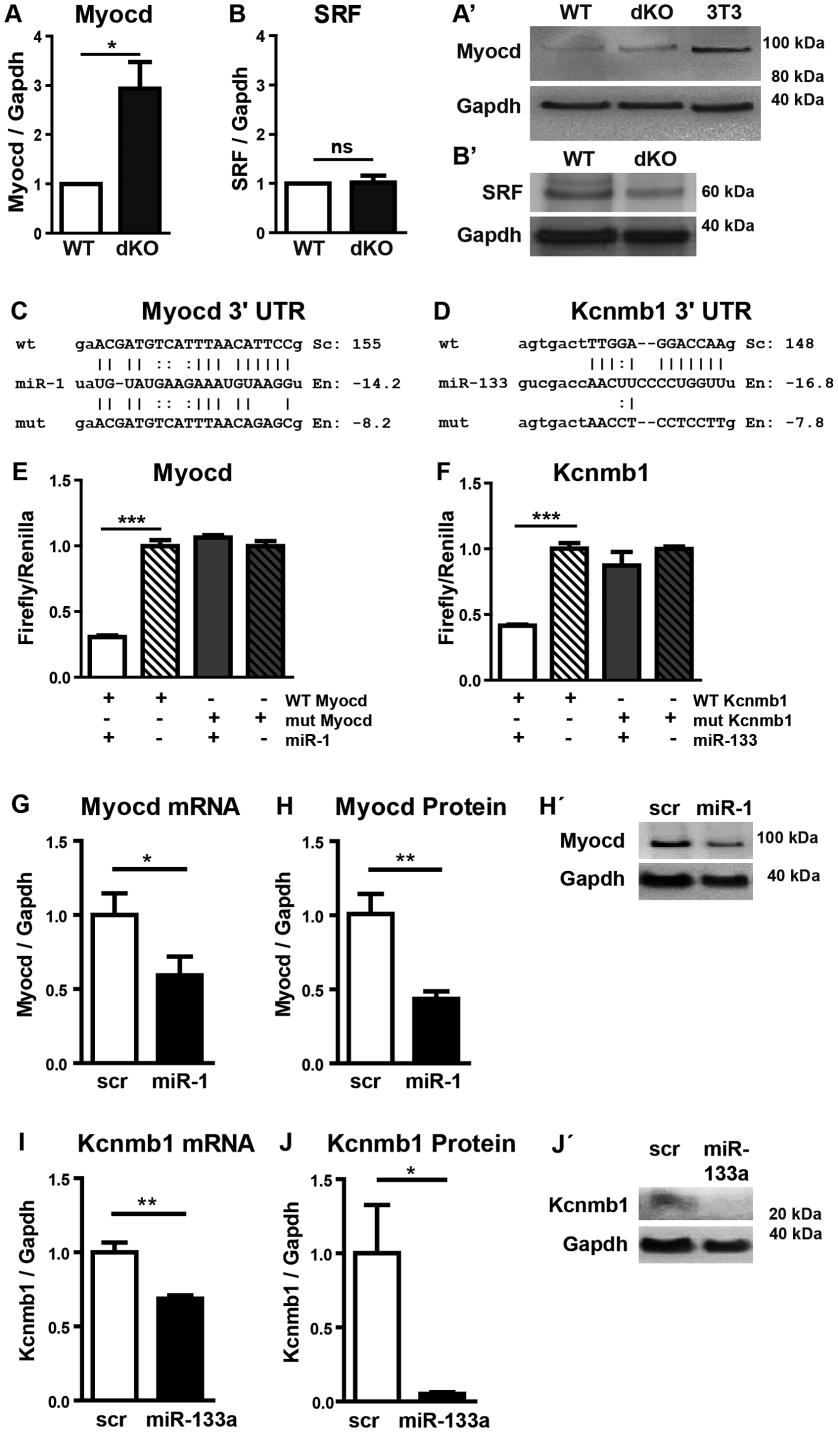
Myocardin is a primary target of miR-1 in the embryonic heart. (A, A′) Western blot analysis of increased myocardin expression in *miR-1/133a* dKO embryonic hearts at E10.5 compared to WT. (B, B′) No increase of SRF protein expression in *miR-1/133a* dKO embryonic hearts at E10.5 compared to WT. (C, D) Putative miR-1 (C) and mir-133a (D) WT and mutant binding sites located in the 3′ UTRs of myocardin and Kcnmb1 mRNAs were cloned into luciferase reporter vectors. (E, F) miR-1 (E) and miR-133a (F) mediated suppression of luciferase activity via WT but not mutant miRNA binding sites located in myocardin (E) and (F) Kcnmb1 mRNAs. Embryonic cardiomyocytes were isolated from embryonic hearts (E11.5-13.5) (G, H, H′). Transfection of miR-1 or of scrambled control (scr) into embryonic cardiomyocytes confirms miR-1 mediated repression of endogenous myocardin transcripts (qRT-PCR; G) and of myocardin protein (Western Blot; H, H′). (I, J, J′) Transfection of miR-133a into embryonic cardiomyocytes confirms miR-133a mediated repression of Kcnmb1 mRNA (qRT-PCR; I) and Kcnmb1 protein (Western Blot; J, J′).

In principle, loss of miRNAs should lead to increased abundance of target transcripts. Hence, we screened for predicted target sites of miR-1 and miR-133a in transcripts that were up-regulated in *miR-1/133a* dKO mutant hearts using Targetscan (v6) and miRanda (microrna.org). 27 out of 382 genes, which were up-regulated at least 1.5-fold, contained conserved target sites for miR-1 or miR-133a. One of the strongest up-regulated genes in that group was myocardin, which carries a conserved target site for miR-1 in the 3′-UTR (Fig. 5C). Myocardin exists in different variants resulting from alternative splicing in cardiomyocytes and smooth muscle cells [22]. Analysis of the expression of different myocardin splice isoforms in E10.5 dKO hearts revealed that only the cardiac-specific but not the smooth muscle-specific isoform of myocardin was up-regulated in dKO hearts essentially ruling out effects of miR-1 on myocardin mRNA splicing (Suppl. Fig. S7). Furthermore, these results indicated that increased abundance of myocardin transcripts is due to miR-1 mediated repression and not caused by general up-regulation of the smooth muscle program.

We also detected a conserved miR-133a target site in the 3′-UTR of Kcnmb1 [23] (Fig. 5D), which is normally specifically expressed in smooth muscle cells but up-regulated in *miR-1/133a* dKO mutant hearts. In contrast, we did not observe transcriptional up-regulation of a number of previously described miR-1 or miR-133a target molecules like SRF, IRQ5, Hand2 or HDAC4 in dKO hearts (Fig. 4). At the protein level, myocardin was 3-fold more abundant in dKO mutants than in wild type controls as indicated by western blot analysis of pools (n = 3) of E10.5 WT and dKO hearts (>4 hearts per pool) (Fig. 5A). The putative miR-133a targets SRF (Fig. 5B, B′) and Hand2 (Suppl. Fig. S8) were not up-regulated at the protein level, which corresponds to the transcriptional analysis. Taken together our results suggested that myocardin represents a primary target for miR-1 and Kcnmb1 for miR-133a miRNAs in vivo at E10.5.

To validate the regulatory interactions between miR-1 and myocardin or miR-133a and Kcnmb1 we inserted the respective miRNA binding sites as well as mutant target sites into the 3′-UTR of a luciferase reporter (Fig. 5C, D). Co-transfection of either miR-1 or miR-133a together with corresponding reporter plasmids efficiently suppressed luciferase activity whereas reporter plasmids carrying mutated miRNA binding sites were not affected (Fig. 5E, F) confirming our assumption that myocardin and Kcnmb1 are primary targets of miR-1 and miR-133a, respectively. To further validate these findings, we transfected miR-1, miR-133 or control miRNA into isolated embryonic cardiomyocytes. As expected, miR-1 overexpression resulted in a significant reduction of myocardin mRNA (Fig. 5G) and protein (Fig. 5H) while miR-133a overexpression caused a significant decline of Kcnmb1 mRNA (Fig. 5I) and protein (Fig. 5J) concentrations compared to miRNA controls (Fig. 5H′, J′).

### Directed expression of myocardin in the heart recapitulates the *miR-1/133a* knock-out phenotype

Myocardin is a potent transcriptional co-activator of serum response factor (SRF) controlling gene expression of smooth muscle and cardiac cells. Disruption of the *myocardin* gene abrogates smooth muscle gene expression during embryonic development and causes programmed cell death in postnatal cardiomyocytes [24], [25]. In addition, overexpression of myocardin in adult cardiomyocytes and other cell types leads to activation of smooth muscle cell genes [26] indicating that a tight regulation of myocardin is necessary for normal heart development. Furthermore, immature cardiomyocytes show several characteristics of smooth muscle cells, such as the expression of smooth muscle marker genes, until approximately E10, which are only lost at later developmental stages when cardiomyocytes mature [2]–[4]. Interestingly, the adverse effects of the loss of miR-1/133a and up-regulation of myocardin became apparent at the same developmental stage when smooth muscle gene expression is normally lost in cardiomyocytes.

**Figure 6:**
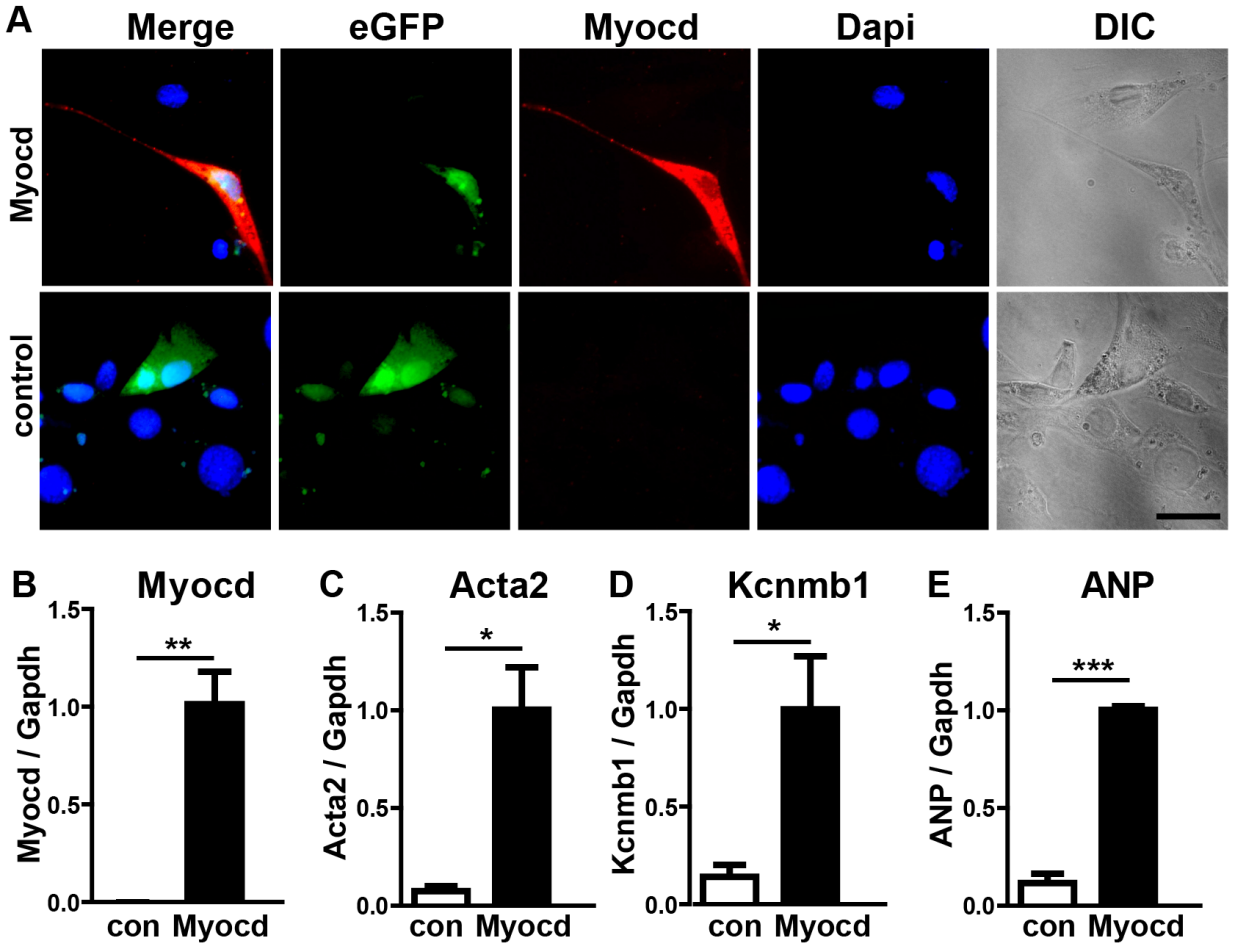
Expression of the miR-1 target myocardin induces smooth muscle cell-like morphology in NIH3T3 cells. (A) An immunofluorescence staining for myocardin is shown. Transfected cells are labeled by EGFP-fluorescence. The scale bar corresponds to 50 µm. (B–E) Quantitative RT-PCR expression analysis of myocardin (B), smooth muscle actin (C), Kcnmb1 (D), and ANP (E) in transfected NIH3T3 cells using Taqman probes.

**Figure 7:**
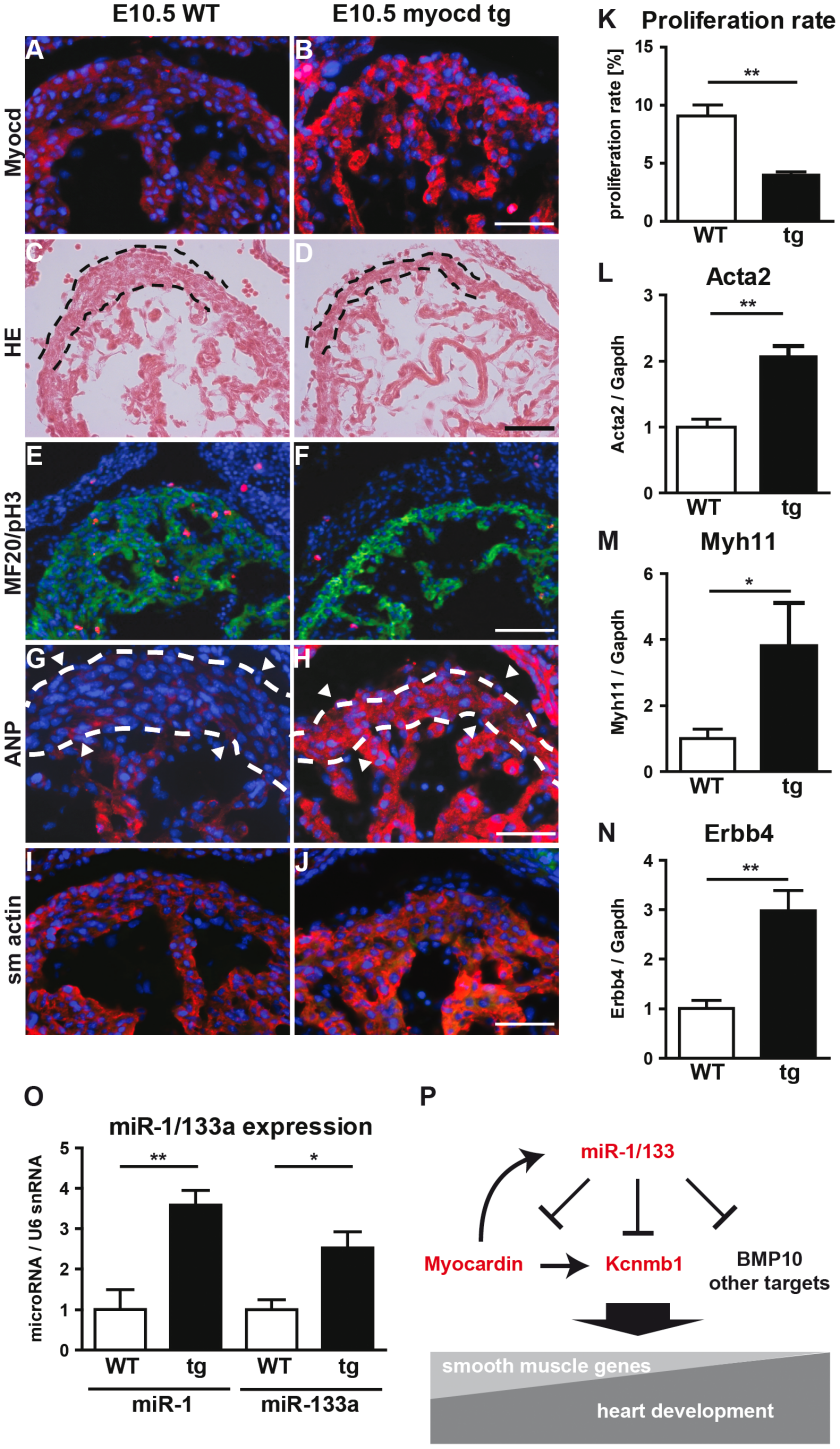
Transgenic overexpression of myocardin in the embryonic heart recapitulates the miR-1/133a phenotype. (A, B) Immunofluorescence analysis of myocardin expression in *myocardin* transgenic and control embryonic hearts at E10.5. (C, D) Morphological analysis of *myocardin* transgenic and control embryonic hearts at E10.5 using H&E stained sections. The reduced diameter of the compact layer of *myocardin* trangenic embryos is clearly visible. (E, F) Immunofluorescence staining for the cardiomyocyte marker MyHC and the proliferation marker phospho-histone 3 (pH3) in *myocardin* transgenic and WT hearts. (G, H) Immunofluorescence staining of increased ANP expression in *myocardin* trangenic hearts. (I, J) Immunofluorescence staining of increased smooth muscle actin expression in *myocardin* transgenic hearts. The scale bar in (B) corresponds to 100 µm in (B, F, H, J), the scale bar in (D) corresponds to 50 µm. (K) Quantitative evaluation of proliferating cardiomyocytes in *myocardin* transgenic and WT hearts. (L–N) Quantitative RT-PCR analysis of increased smooth muscle actin (Acta2) (L), Myh11 (M), and Erbb4 (N) in *myocardin* transgenic compared to WT hearts. (O) Quantitative RT-PCR analysis of increased expression of miR-1 and miR-133a in *myocardin* transgenic E10.5 embryonic hearts. (P) Model illustrating the negative feedback loop controlling expression of miR-1/133a, myocardin and Kcnmb1.

To understand the molecular events leading to the *miR-1/133a* dKO phenotype and to analyze whether increased levels of myocardin induce a similar set of genes up-regulated in *miR-1/133a* dKO cells, we decided to overexpress myocardin *in vitro* in NIH3T3 cells and *in vivo* in embryonic hearts. Myocardin overexpressing NIH3T3 cells, marked by IRES mediated co-expression of EGFP, acquired a SM-cell like spindle shaped morphology and started to express SM-specific genes confirming previous results (Fig. 6A–E) 27,28. Affymetrix GeneArray and quantitative RT-PCR analysis revealed an up-regulation of smooth muscle marker genes including Acta2 and Kcnmb1, which resembled several of the transcriptional changes observed in *miR-1/133a* KO hearts (Fig. 6B–E, Suppl. ). Remarkably, overexpression of myocardin expression even stimulated expression of ANP in NIH3T3 cells (Fig. 6E). Next, we generated mice overexpressing myocardin via the heart-specific *α-MHC* promoter (Fig. 7A, B). Since overexpression of myocardin in the heart resulted in early embryonic lethality we used F0 embryos, newly generated for each individual experiment. Transgenic embryos (n = 11) with similar levels of myocardin mRNA in individual embryonic hearts were used for further analysis. Hearts of myocardin-expressing transgenic embryos showed a thin compact layer and a preserved trabecular structure at E10.5 (Fig. 7C, D) strongly resembling the morphologic phenotype seen in *miR-1/133a* dKO embryos. We also observed reduced proliferation of cardiomyocytes at E10.5 (Fig. 7E, F, K) and ectopic expression of ANP in the remaining compact layer (Fig. 7G, H). Normally, expression of ANP is confined to the trabecular layer at this developmental stage.

Transcriptome analysis of *myocardin* transgenic hearts revealed additional similarities between *miR-1/133a* dKO and *myocardin*-transgenic hearts. Interestingly, these similarities were not only restricted to up-regulation of smooth muscle-marker genes such as Acta2, Myh11 (Fig. 7L, M) but also included dysregulation of other genes involved in heart development (*Erbb4*, *BMP2*, *BMP-7*, *Myo18b*, *Akap2*, *Palm2*, *Ppargc1a*, *Cacna1d*, *Cacnb2*) (Fig. 7N, Suppl. Fig. S10). Most importantly, we observed a striking overlap of genes up-regulated in myocardin-transgenic and *miR-1/133a* dKO hearts. 90 out of 139 genes up-regulated by 1.5-fold in *myocardin*-transgenic hearts were also up-regulated in *miR-1/133a* dKO mutants (Suppl. Fig. S10A) providing a convincing molecular explanation for the similarity of *miR-1/133a* dKO and *myocardin*-transgenic heart phenotypes. While the majority of dysregulated genes in *miR-1/133* KO mice and myocardin overexpressing mice showed an up-regulation (Suppl. Fig. S10B) we found only few genes that were down-regulated both in *miR-1/133* dKO embryos and in myocardin overexpressing embryos. This observation is in line with the established function of the transcriptional coactivator myocardin suggesting that increased myocardin levels primarily stimulate transcriptional activity. In contrast, it seems likely the down-regulation of Msx1/2 [29] and Tbx1/Six1/Eya1 [21] in *miR-1/133a* dKO hearts occurred by secondary means independent of the up-regulation of myocardin, probably due to the loss of myocardial cells or the global arrest of heart development in *miR-1/133a* dKO mutants. Of note, we did not observe increased BMP-10 expression in myocardin overexpressing E10.5 hearts as in *miR-1/133a* dKO mutants (Suppl. Fig. S10). Since, we found a miR-1 binding site located within the ORF region of BMP-10 mRNA, which is able to repress BMP-10 mRNA as indicated by luciferase reporter assays *in vitro*, it seems likely that miR-1 directly represses BMP-10 *in vivo* (Suppl. Fig. S11) and thereby normalizes BMP-10 levels in *myocardin* transgenic embryos. Our analysis suggested that the loss of miR-1 mediated repression of myocardin initiates a cascade of molecular events that is responsible for many aspects of *miR-1/133a* dKO phenotype.

### Myocardin regulates miR-1/133 expression *in vivo*

**Figure 8:**
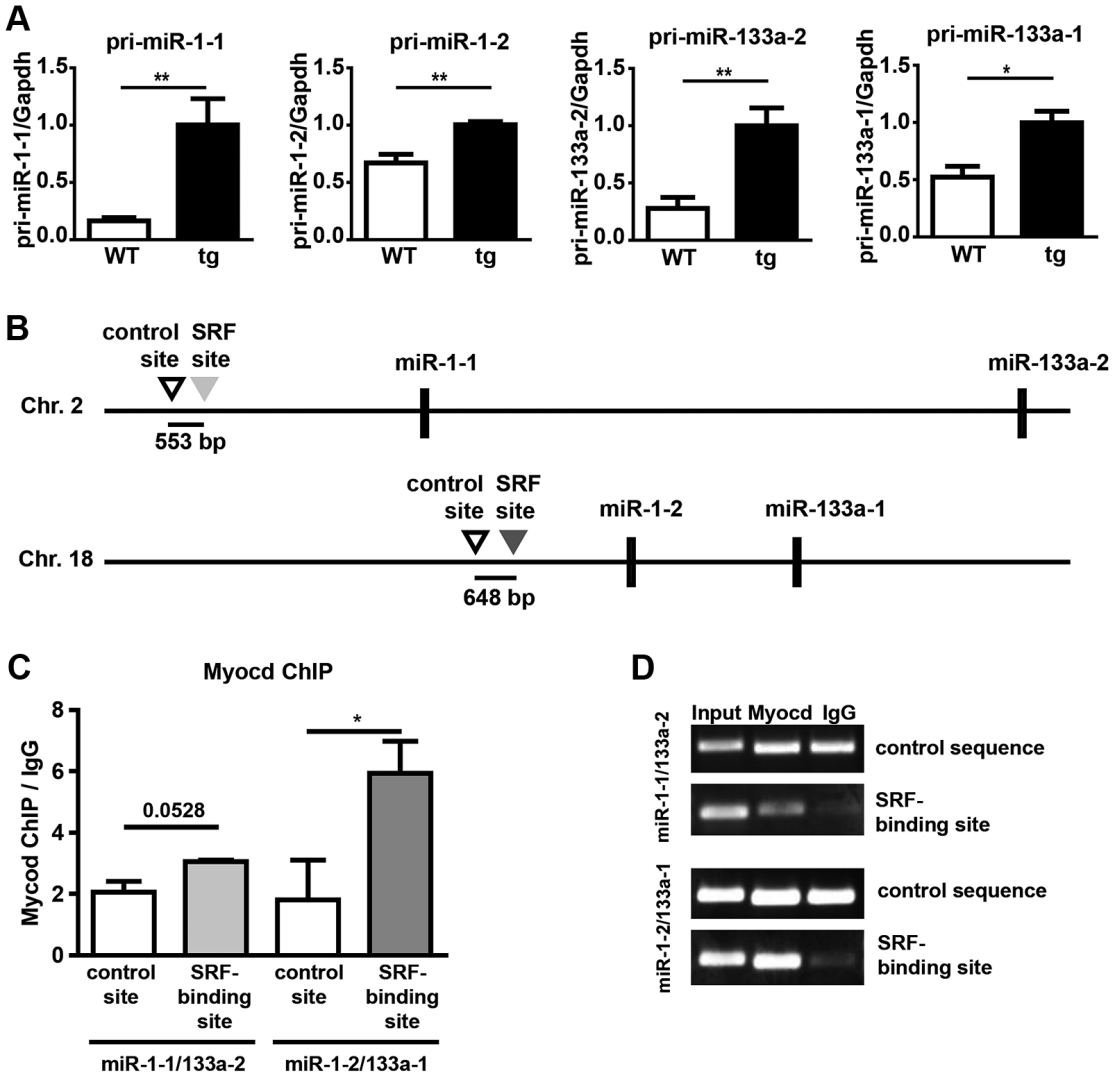
Increased expression of pri-miR-1/133a in Myocardin overexpressing embryos and analysis of the interaction of myocardin with SRF-binding sequences in miR-1-1/133a-2 and miR-1-2/133a-1 promoters. (A) qRT-PCR expression analysis of pri-miR1-1, pri-miR1-2, pri-miR133-a1 and pri-miR133a-2 in Myocardin overexpressing embryonic hearts. Overexpression of myocardin leads to up-regulation of miR-1 and miR-133a. Taqman probes specific for individual pri-miRNAs representing primary unprocessed transcripts of either the miR-1-1/133a-2 or the miR-1-2/133a-1 gene were used for amplification. (B) Schematic representation of the location of SRF-binding sites and control sequences in miR-1-1/133a-2 and miR-1-2/133a-1 clusters. (C) Chromatin immunoprecipitation using anti-myocardin antibodies demonstrates binding of myocardin to an SRF-site (bs) 5′ of the miR-1-2/133a-1 cluster but not to a SRF-site in the miR-1-1/133a-2 cluster. Sequences within respective clusters not carrying CArG motifs (con) were used as controls. (D) Myocardin ChIP qRT-PCR products analyzed by gel electrophoresis. Results of PCRs for input, myocardin ChIP and IgG control ChIP are shown.

Previous studies demonstrated that *miR-1* genes are direct transcriptional targets of the transcription factor SRF [14], which depends on myocardin or MRTFs to achieve cell type specific transcriptional activity [24]. Since we demonstrated that miR-1 represses myocardin we speculated that miR-1 might be part of a negative feedback loop that restricts its own expression. Therefore, we analyzed expression of miR-1 in *myocardin* transgenic hearts at E10.5. Interestingly, we found a strong induction of mature miR-1/133a levels (Fig. 7O). Additional RT-PCR based Taqman assays designed to detect pri-miR-1-1, pri-miR-1-2, pri-miR-133a-2, and pri-miR-133a-1 unveiled increased expression of all pri-miRNAs (Fig. 8A) indicating that both *miR-1/133a* clusters are activated by myocardin. To investigate whether myocardin activates the *miR-1-1/133-a2* and the *miR-1-2/133-a1* gene clusters via previously mapped SRF binding sites [14], [30], we performed chromatin immunoprecipitation (ChIP) experiments in C2C12 muscle cells using myocardin antibodies. We found that myocardin bound strongly to the SRF binding site up-stream of the *miR-1-2/133a-1* gene cluster on chromosome 18 but not to the site up-stream of the *miR-1-1/133a-2* gene (Fig. 8B, C) suggesting control of *miR-1-2/133a-1* expression by a ternary complex composed of SRF and myocardin. In contrast, the *miR-1-1/133a-2* gene might be regulated via a so far undisclosed SRF binding site or by other transcription factors such as MEF2 [10] or Tbx5 [7] that are co-activated by myocardin. Taken together, our results suggest that miR-1 mediated repression of myocardin limits transcriptional activation of both *miR-1* and *miR-133a* clusters thereby adjusting its expression (and of *miR-133a*) in a negative feedback loop (Fig. 7P).

## Discussion

The presence of multiple miRNAs with identical or similar mature sequences is a common and evolutionary conserved feature in several species indicating functional significance for the presence of several similar or identical miRNA genes [31]. miR-1 and miR-133a represent a particularly intriguing example since the two gene clusters, which encode mir-1-1/133a-2 and miR-1-2/133a-1, are completely identical and apparently expressed in the same tissue: heart and skeletal muscle [6], [7]. The lack of gross morphological abnormalities after genetic inactivation of single *miR1-1/miR-133a* gene cluster mutants seems to indicate redundant functions but does not rule out a differential requirement of individual *miR-1/133a* gene clusters under specific conditions. In fact, we found that inactivation of *miR-1-1/133a-2* but not *miR-1-2/133a-1* impaired the ability of the heart to maintain a physiological ejection fraction after TAC-induced pressure overload. It seems likely that other, so far unknown conditions might predominantly require activity of the *miR-1-2/133a-1* gene cluster.

The lack of developmental abnormalities in single *miR-1/133a* gene cluster mutants corroborates previous findings on *miR-133a-1* and *miR-133a-1* KO animals [15]. In contrast, deletion of *miR-1-2* has been reported to cause incompletely penetrant developmental and electrophysiological phenotypes [16], which do not fit to our observations. In principle, it is possible that the concomitant deletion of both *miR-1-2* and *miR-133a-1* rescues a potential phenotype caused by the inactivation of *miR-1-2* alone but it seems more likely that deletion of *miR-1-2*, which is located in an intron of the *mib1* gene and close to the transcriptional start-site of *RP24-66N1*, a non-coding antisense transcript, has affected regulation of neighboring genes [32], [33]. Alternatively, the remaining *miR-1-1* gene might be expressed at lower levels on the genetic background used by Zhao et al. [16] thereby compromising its compensatory activity and causing an incompletely penetrant phenotype.

Deletion of both *miR-1/133a* clusters revealed a fundamentally new role of miR-1/133a in early heart development. The *miR-1/133a* dKO phenotype differs significantly from the previously described defect of *miR-133a* dKO mice, which becomes apparent only at later stages [15] suggesting fundamentally different mechanisms. The complete loss of miR-1/133a did not interfere with formation of the primary heart tube but affected maturation and further specification of embryonic cardiomyocytes during expansion of the compact layer of the myocardium. We observed that *miR-1/133a* dKO cardiomyocytes failed to get rid of their hybrid smooth muscle/cardiomyocyte phenotype and did not acquire a more mature cardiomyocyte-specific identity. Unbiased transcriptional profiling and molecular analysis of putative miR-1/133a target molecules up-regulated in *miR-1/133a* dKO mutants uncovered several direct targets of miR-1 and miR-133a including myocardin, Kcnmb1 and BMP-10. We reasoned that the up-regulation of myocardin, which is a well-characterized transcriptional co-activator of SRF that serves as a major regulatory switch for smooth muscle gene expression [24], [27], is instrumental for mediating effects of miR-1 during early heart development. In fact, we discovered that miR-1 directly regulates myocardin mRNA in a heterologous expression system via a target site in the 3′ UTR using luciferase reporter assays and demonstrated that over-expression of miR-1 in embryonic cardiomyocytes resulted in a down-regulation of myocardin.

Myocardin plays an important role for the development of visceral and vascular smooth muscle cells. Initially, its function in cardiomyocytes remained unknown due to the lack of a cardiac phenotype in constitutive myocardin *knock-out* mice [34]. However, more recent studies using cardiac specific deletions of myocardin demonstrated that myocardin is required for cardiomyocyte survival and maintenance of heart function after birth [25], [35]. At E9.5, *myocardin* mutant hearts show a pronounced reduction of cardiomyocyte proliferation, which was explained by the inability of SRF to up-regulate BMP-10 in the absence of myocardin [5]. Interestingly, *ex vivo* culture of *myocardin* mutant hearts in BMP-10 conditioned media rescues cardiomyocyte proliferation suggesting a pivotal role of BMP-10 in the control of cardiomyocyte proliferation in embryonic hearts. The reduction of cardiomyocyte proliferation in *miR-1/133a* dKO seems to rely on a different mechanism since BMP-10 expression was increased in *miR-1/133a* dKO mice but not decreased as in *myocardin* mutants [5]. We assume that the failure of immature *miR-1/133a* dKO mutant cardiomyocytes to acquire a more mature phenotype activates a cellular stress program inhibiting further proliferation, since no evidence for direct regulation of cell proliferation by miR-1/133a was found.

The link between adjusted myocardin expression levels and expression of smooth muscle marker genes is evident in various pathological conditions of the heart, which are characterized by concomitant up-regulation of myocardin and smooth muscle markers [26], [36]. To explain the fact that myocardin induces smooth muscle genes in various cells but fails to do so in cardiomyoyctes of healthy fetal and adult hearts, has been explained by the existence of putative negative regulators neutralizing myocardin or of additional cofactors required for myocardin activity in cardiomyocytes [24]. Our results suggest that miR-1 is one of the postulated negative factors restricting myocardin activity in cardiomyocytes. Down-regulation of miR-1, which occurs under different pathological conditions [10], and subsequent increase of myocardin activity might explain the up-regulation of smooth muscle marker genes in various diseases of the heart [5].

Initially, it was surprising to see that the increased myocardin activity in *miR-1/133a* dKO mutants and *myocardin* transgenic embryos caused up-regulation of multiple smooth muscle marker genes, since the relatively normal expression of smooth muscle genes in *myocardin* mutant hearts seems to suggest that smooth muscle gene expression in cardiomyocytes is not under direct control of myocardin [34], [35]. Yet, expression of the myocardin-related genes MRTF A and B might substitute for the lack of myocardin in the heart [35]. Furthermore, increased myocardin expression might stimulate expression of smooth muscle genes even if basal activity of smooth muscle genes under physiological conditions is controlled by other means (i.e. MRTF A and B).

Transgenic overexpression of myocardin in the developing heart, which closely mimicked the transcriptional increase seen in *miR-1/133a dKO* mice, recapitulated many aspects of the *miR-1/133a* dKO phenotype proving the mechanistic relevance of myocardin up-regulation. Specifically, myocardin overexpression phenocopied morphological changes, reduced cardiomyocyte proliferation, and induced expression of a large set of smooth muscle marker genes all observed in *miR-1/133a* dKO mutants. In total, 90 out of 139 genes up-regulated by myocardin overexpression were also up-regulated in *miR-1/133a* dKO mutant hearts. Of course, up-regulation of myocardin does not account for all effects of miR-1/133a as illustrated by 382 genes up-regulated in *miR-1/133a* dKO mutants but not in myocardin overexpressing hearts. For example, expression of BMP-10 was not increased in *myocardin* transgenic embryos. The missing up-regulation of BMP-10 in myocardin-overexpressing embryos does not argue against the control of BMP-10 transcription by myocardin, which has been recently demonstrated using *myocardin* mutant embryos [5]. Rather, it supports the relevance and the efficiency of miR-1 mediated repression of BMP-10, which seems to be able to normalize increased BMP-10 levels in myocardin overexpressing mice. Surprisingly, we did not observe up-regulation of several previously described miR-1/133 target mRNAs such as SRF, IRQ5, Hand2 or HDAC4 in *miR-1/133* dKO mutant hearts at E10.5 [6], [7]. Apparently, stage-specific, context-dependent mechanisms restrict inhibitory effects of miRNAs probably by blocking access of miRNAs to certain targets or by potential secondary compensatory effects.

Transgenic overexpression of myocardin in the heart resulted in a strong induction of expression of both *miR-1/133* gene clusters, which together with the inhibition of myocardin by miR-1 suggests the existence of a negative regulatory loop that acts as a rheostat to regulate *miR-1/133a*. Intriguingly, the genetic linkage of *miR-1* and *miR-133a* allows concomitant regulation of both genes by myocardin thereby including *miR-133a* into the negative feedback loop constituted by miR-1 and myocardin. It is tempting to speculate that the joint regulation of two different miRNA genes targeting different genes by myocardin is a reason for the evolutionary conservation of *miR-1* and *miR-133a* linkage. The miR-133a target Kcnmb1 that is controlled at the transcriptional level by myocardin is another component of this regulatory network. miR-133a-mediated inhibition of Kcnmb1 and miR-1-mediated suppression of myocardin render Kcnmb1 expression dependent on the balance of miR-1/133a and myocardin concentrations in the cell, which might be important for the regulation of pathophysiologic conditions. Myocardin seems to exert its regulatory activity on *miR-1-2/133a-1* via SRF since myocardin was detected by ChIP at a SRF site in the *miR-1-2/133a-1* promoter. We did not detect binding of myocardin to the SRF-binding CArG box in the *miR-1-1/133a-2* promoter. It is possible that additional CArG elements that have a functional impact on the expression are located further upstream or in intronic regions, which were not included in the analysis. Alternatively myocardin might also regulate the *miR-1-1/133a-2* promoter via MEF-2 and Tbx5, which are also bound and co-activated by myocardin [7], [10]. Genome wide ChIP-seq experiments will probably solve this question in the future. Taken together our findings illustrate the complex nature of miRNA-mediated regulatory processes, which spatially and temporally restrict gene activity thereby allowing transcriptional co-activators such as myocardin and other effectors to act in a stage-specific manner.

## Materials and Methods

### Ethics statement

All animal experiments were in accordance with German animal protection laws and were approved by the local governmental animal protection committee.

### *Knock out*, transgenic mice and cell culture

The *miR-1-1/133a-2* genomic region was deleted by homologous recombination with a targeting vector inserting an IRES-lacZ-neomycin resistance cassette into the NdeI site of pre-miR-1-1 deleting the *miR-1-1* and *miR-133a-2* coding regions down to the BamHI site located 140 bp 3′ of *miR-133a*. The vector contained 4 kb 5′ and 6 kb 3′ homologous 129 genomic sequence. The *miR-1-2/133a-1* genomic region was deleted by homologous recombination with a targeting vector that replaced the genomic region coding for pre-miR-133a to pre-mir-1-2 with the IRES-LacZ-neomycin cassette. The vector contained 3 kb genomic region flanking pre-mir-1-2 at the 5′ and 3.5 kb genomic sequence flanking pre-miR-133a at the 3′. Both targeting vectors contained a DTA cassette for negative selection. Targeting vectors were electroporated into MPIII 129SV embryonic stem cells. Homologous recombination was confirmed by Southern Blot analysis using appropriate restriction endonucleases (suppl. info1) and probes hybridizing to genomic regions outside of the vector. Recombinant ES cells were injected into blastocysts and transferred to pseudopregnant mice. Chimeric mice were backcrossed to C57Bl/6 mice. Heterozygous animals were mated to obtain homozygous animals. For ectopic myocardin expression, the myocardin ORF representing splice form NM_145136.4 obtained from shuttle clone OCACo5052D0518D (BioScience) was inserted into the BamHI site of pIRES2-EGFP (Clontech). The CMV-promoter containing pIRES2-EGFP-pA based construct was used to transfect proliferating NIH3T3 cells (ATCC) with Lipofectamine 2000 (Invitrogen) according to the manufacturer's instructions. The myocardin-IRES2-EGFP-pA cassette was inserted 3′ to 5.6 kb of the murine *Myh6* promoter (*cardiac α-MHC*). Transgenic embryos were newly generated for each individual experiment by pronuclear injection using the Myh6-myocardin-IRES2-EGFP-pA fragment following standard procedures. Recovered embryos were examined for GFP expression and genotyped by PCR using transgene-specific primers (ACATTGCCAAAAGACGGCAATATGG, GGAATGGCTGGACCTCACTCCACCTAG) using extraembryonic tissue. All transgenic animals used for further analysis (n = 11) expressed similar levels of myocardin as measured by qRT-PCR.

### Microarrays and quantitative RT-PCR

Total RNA from whole hearts of E10.5 embryos or NIH3T3 cells was isolated using the Trizol method (Invitrogen). RNA quality was verified using the Agilent Bioanalyser and the RNA 6000 Nano Kit. RNA was labeled following the protocol of Affymetrix. Labeled samples were hybridized to Affymetrix GeneChip Mouse Gene 1.0 ST arrays, processed, scanned and analyzed (RMA with Affymetrix Expression console, statistical analysis using Student's t-test with DNAStar Arraystar 5.0). Enrichment of GO annotation and generation of Venn diagrams were accomplished using DNAStar Arraystar 5.0. TaqMan Gene Expression Assays were used for quantitative RT-PCR analysis employing the Applied Biosystems StepOnePlus system. The following FAM dye TaqMan assays were used (BMP-10: Mm01183889, Nppa: Mm01255748_g1, Myocd: Mm01325105_m1, Erbb4: Mm01256793_m1, Myh11: Mm00443013_m1, Acta2: Mm01204962_gH, Tgln1: Mm00441661_g1; Applied Biosystems). For normalization, VIC labeled Gapdh assay was used (ID 4352339E). Expression of miRNAs was quantified using FAM labeled TaqMan microRNA Assays (miR-1: #002222, miR-133a: #002246). TaqMan MicroRNA Reverse Transcription Kit (#4366596) was applied to convert miRNA to cDNA. VIC labeled TaqMan Assays detecting U6 snRNA (#001973) were used for normalization. Relative expression was calculated using the ΔΔCt method. Kcnmb1 expression was analyzed by qRT-PCR using specific oligonucleotides (CTGGGAGTGGCAATGGTAGTG, CCGAGTGTCTTCCGTGTGATAC, as described previously [23]. The RT-PCR was performed using a FastStart Universal SYBR-Green Mastermix (Roche) to allow quantification. Data were normalized to Gapdh detection (ACCACAGTCCATGCCATCAC, CATGCCAGTGAGCTTCCCGT).

### Pri-microRNA RT-PCR

Precursor microRNAs of miR-1-1/miR-133a-2 or miR-1-2/miR-133a-1 were detected using taqman assays specific for pri-miR-1a-1 (m18), pri-miR-1a-2 (m19), pri-miR-133a-1 (m20) and pri-miR-133a-2 (m21) following instructions of the manufacturer.

### Chromatin immunoprecipitation

Proliferating C2C12 cells (ATCC) were treated with 1% formaldehyde/PBS for 10 minutes at room temperature before termination of the reaction by addition of 125 mM glycine. Cells were washed using PBS and incubated with cell lysis buffer (5 mM HEPES pH 8, 85 mM KCl, 0.5% NP-40, protease inhibitor mix) for 10 minutes. Nuclei were isolated by centrifugation (5000 rpm, 5 min) and incubated with nucleus lysis buffer (50 mM Triscl pH 8.1, 10 mM EDTA, 1% SDS, protease inhibitor mix) at 4°C for 10 minutes. DNA was fragmented to an average fragment length of 200–500 bp using sonication (Bioruptor, Diagenode). The DNA was diluted to 500 ng/µl in dilution buffer (0.01% SDS, 1.1% Triton X-100, 1.2 mM EDTA, 16.7 mM TrisCl pH 8.1, 167 mM NaCl) and incubated with Protein A Agarose beads. 5% of the supernatant was kept as total input control. 1 µg of DNA were incubated with anti-myocardin antibody (R&D systems) or with IgG control (Diagenode) at 4°C overnight. 25% of antibody-binding beads (KCH-503-008, Diagenode) were added to samples and incubated 2 hours at 4°C. Subsequently, beads with bound complexes were consecutively washed with low salt buffer (0.1% SDS, 1% Triton X-100, 2 mM EDTA, 20 mM TrisCl pH 8.1, 150 mM NaCl), high salt buffer (0.1% SDS, 1% Triton X-100, 2 mM EDTA, 20 mM TrisCl pH 8.1, 500 mM NaCl), LiCl buffer (10 mM TrisCl pH 8, 250 mM LiCl, 1% NP-40, 0.5% deoxycholic acid, 1 mM EDTA) and TE buffer. Next, 10% Chelex (Biorad) was added to the beads and to the input. Samples were incubated with Proteinase K at 37°C for 30 minutes after incubation at 95°C for 10 minutes. ChiP samples were incubated again at 95°C for 5 min and centrifuged. The supernatant was used for qRT-PCR using SYBR Green Mastermix (Roche) with specific primers (miR-1-2/133a-1: fwd TTGCTTTGGGATTCTTTTGG, rev TCGGGAAGAACATAGGTTGG, miR-1-2/133a-1 control: fwd CCCAGCAAATCTATAAAGA, rev GCCTGTGTGAGGTGATATAG, miR-1-1/133a-2: fwd ATACAACCCAGGTGGGAACA, rev AGAATTGCAGGTCACCTTGG, miR-1-1/133a-2 control: fwd GTGAGGACAGATTAGCCAGTAC, rev CTTCAAGCTCCTCAGAAGGC with an annealing temperature of 60°C following the manufactures instructions.

### Whole mount in situ hybridization

Whole mount in situ hybridization for miRNAs was performed as described previously [11] using dual DIG-labeled LNA antisense probes (Exiqon) for mmu-miR-1.

### Morphological analysis

Embryos of different developmental stages were isolated and immediately fixed in PFA. Tissues of postnatal animals were isolated after transcardial PFA perfusion. For paraffin sections, samples were dehydrated following standard protocols, embedded into paraffin and sectioned at 10 µm and H&E stained. For cryosections, tissues were equilibrated in 30% sucrose/PBS, frozen on dry ice. 10 µm sections were mounted on Superfrost slides.

### Proliferation assay

Pregnant mice were injected i.p. with 3 mg EdU (5-ethynyl-2′deoxyuridine, Click-iT EdU Imaging Kit, Invitrogen C10339). Embryos were recovered at E9.5 and E10.5, fixed with PFA and embedded for cryosections. EdU staining was performed according to the manufacturer's instructions. Staining for Phospho-Histone H3 was performed as described in the Immunohistochemistry protocol. Nuclei were stained with Dapi (1∶1000), sections were mounted with Fluoromount and analyzed using Zeiss Axioimager (Z1).

### Immunohistochemistry and Western blot analysis

For immunohistochemistry, recovered embryos were fixed in 4% PFA for 2 h and genotyped using extra embryonic tissues. Embryos were incubated for 1 h each in PBS and 15% sucrose followed by an overnight treatment with 30% sucrose. Afterwards, embryos were embedded in TissueTek, cryosectioned, and mounted on Superfrost slides. Sections were fixed in 4% PFA, washed with PBS and incubated in blocking solution containing 5% NGS (normal goat serum), 1% BSA and 0.3% Triton-X100 for 1 h at RT. Antibodies were incubated in blocking solution 1∶200 overnight at 4°C. After washing with PBS, secondary antibodies and DAPI was applied for 1 h at RT, followed by 3×5 min washing in PBS and embedding in Fluoromount. Pictures were taken using a Z1 axioimager (Zeiss).

For Western blot analysis, 10 µg total protein extracts from 3 pools of embryonic E10.5 hearts (n = 4) were loaded on NuPAGE Novex Bis-Tris gels (Invitrogen) and blotted on nitrocellulose membranes. Quantification of the Western blots was performed by densitometry using the Femto-kit (Pierce) and Versadoc-System (Biorad). The following antibodies were used according to the manufacturer's recommendations: anti-Nppa (1∶200, AB 5490, Chemicon), anti-α-Smooth muscle actin (1∶500, Clone 1A4, Cy3 conjugated, Sigma), anti-Myocardin (1∶200, pAB0604 Covalab), anti-Tag(CGY)FP (1∶200, AB121 Evrogen), anti-Myocardin (1∶500, MAB4028, R&D), anti-SRF (1∶500, SC-335, Santa Cruz), anti-Kcnmb1 (1∶200, FL-191, Santa Cruz), anti- Hand2 (1∶200, AF3876, R&D Systems), anti-Phospho-Histone H3 (Ser-10) (1∶200, #32219, Upstate cell signaling solutions), anti-Gapdh (1∶1000, 14C10 Cell signaling). Secondary antibodies were: anti-rabbit-Alexa594 (1∶1000, A11012, Invitrogen), anti-mouse-Alexa594 (1∶1000, A11005, Invitrogen), anti-rabbit-Alexa488 (1∶1000, A11070, Invitrogen), anti-rabbit-Alexa488 (1∶1000, A1101, Invitrogen).

### Luciferase reporter assay

WT and mutated miRNA binding sites were directionally cloned in quadruplicate into the NheI and XhoI sites of the pmirGLO Dual-Luciferase Vector (E13330, Promega) using oligonucleotides. (myocd miR-1 binding site: AGAGAACGATGTCATTTAACATTCCGAGGAGAACGATGTCATTTAACATTCCGAGGAGAACGATGTCATTTAACATTCCGAGGAGAACGATGTCATTTAACATTCCGAGA; myocd mutant miR-1 binding site: AGAGAACGATGTCATTTAACAgagCGAGGAGAACGATGTCATTTAACAgagCGAGGAGAACGATGTCATTTAACAgagCGAGGAGAACGATGTCATTTAACAgagCGAGA; Kcnmb1 miR-133a-binding site: AGAAAGGCCTCCTAGGAGGACCAAGGAGAGAAAGGCCTCCTAGGAGGACCAAGGAGAGAAAGGCCTCCTAGGAGGACCAAGGAGAGAAAGGCCTCCTAGGAGGACCAAGGAG; mutant Kcnmb1 miR-133a-binding site: AGAAAGGCCTCCTCTACTAATCACGGAGAGAAAGGCCTCCTCTACTAATCACGGAGAGAAAGGCCTCCTCTACTAATCACGGAGAGAAAGGCCTCCTCTACTAATCACGGAG). 70%-confluent HEK293 cells were transfected with 50 ng of the respective plasmid/24-well with or without 50 pmol of miRIDIAN microRNA mimic miR-1 or miR-133a (Thermo) using Lipofectamine 2000 (Invitrogen). Each transfection was done in triplicate. Cells were lysed 24 h after transfection. Firefly luciferase and renilla activities were determined using the Dual-Luciferase Reporter assay (Promega) and the Mithras LB940plate reader (Berthold). Firefly luciferase intensities were normalized to Renilla activities.

### MRI measurements

Cardiac MRI measurements were performed on a 7.0 T Bruker Pharmascan, equipped with a 300 mT/m gradient system, using a custom-built circularly polarized birdcage resonator and the IntraGateTM self-gating tool [37]. The parameters for identification of the ECG were adapted for one heart slice and transferred afterwards to the navigator signals of the remaining slices. Thus the in-phase reconstruction of all pictures is guaranteed. MRI data were analyzed using Qmass digital imaging software (Medis). Mice were measured under volatile isoflurane (1.5–2.0%) anesthesia. Measurements were based on the gradient echo method (repetition time = 6.2 ms; echo time = 6.0 ms; field of view = 2.20×2.20 cm; slice thickness = 1.0 mm; matrix = 128×128; repetitions = 100). The imaging plane was localized using scout images showing the 2- and 4-chamber view of the heart, followed by acquisition in short axis view, orthogonal on the septum in both scouts. Multiple contiguous short-axis slices consisting of 7 to 10 slices were acquired for complete coverage of the left and right ventricle.

### Isolation of embryonic cardiomyocytes and transfection

Embryonic hearts (E11.5–13.5, n = 254) were dissected and atria as well as vessels were removed. Remaining ventricles were washed with culture medium (DMEM 4.5 g/ml, 10% FCS, 1× PS, 0.1× NEAA), incubated three times in predigestion buffer (154.6 mM NaCl, 11.1 mM Glucose, 0.027 mM KCl, 0.028 mM NaH_2_PO_4_×H_2_O, 11.9 mM NaHCO_3_, 2.5 g/ml Pancreatin, 9.9 mM 2,3 butanedione monoxine; Sigma B0753) for 5 minutes at 37°C. Samples were incubated in digestion buffer (predigestion buffer containing 0.25 mg/ml Liberase, Roche) 6 to 9 times each time depending on embryonic stage followed by 5 min 1200 rpm centrifugation. Supernatants were pooled and centrifuged, cells were recovered in culture medium and plated on 1% gelatine coated 24 well plates. Medium was changed to medium without antibiotics 12 hours before transfection. Embryonic cardiomyocytes were transfected with 100 pmol miRIDIAN microRNA Mimic (Thermo Scientific) and 1.8 µl of DharmaFECT 2 transfection reagent (Thermo Scientific) according to manufactures instructions. Total RNA and protein was isolated 24 hours after transfection.

### Statistical analysis and accession numbers

Statistical analysis of Western blot, RT-PCR, cell proliferation and reporter genes assays was performed using Student's t-test. p-values <0.05 were considered to be significant. Microarray data are available via arrayexpress hosted by the EBI (http://www.ebi.ac.uk/arrayexpress/). Accession numbers are “E-MEXP-3869” and “E-MEXP-3873.”

## Supporting Information

Figure S1 Deletion of miR-1/133a coding clusters on mouse chromosome 2 and chromosome 18. The genomic regions coding for miR-1-1/133a-2 on mouse chromosome 2 and miR-1-2/133a-1 on mouse chromosome 18 were replaced with loxP-flanked IRES-LacZ/neoR cassettes. Recombination of the genomic locus was analyzed using probes located at genomic regions outside of the respective targeting vectors. Localization of the Southern blot probes is indicated. Both mouse lines were bred to generate homozygous offspring.
(TIF)

Figure S2 Deletion of single miR-1/133a genomic clusters does not lead to gross morphological alterations in heart and skeletal muscle. (A) HE stained transverse sections of the ventricle reveal no histological abnormalities. The scale bar in (A) corresponds to 50 µm. (B) Immunofluorescence analysis of skeletal muscles of miR-1-1/mirR-133a-2 and miR-1-2/mirR-133a-1 homozygous mutant mice. No increase of cellularity, centrally located nuclei indicating regeneration or changes in the diameter of myotubes are visible on cross sections stained with Triticum vulagaris lectin and DAPI. The scale bar in (B) corresponds to 100 µm.
(TIF)

Figure S3 Deletion of both miR-1/133a clusters leads to arrest of heart development and embryonic lethality. (A–D) Macroscopic views of isolated wild type (WT) and miR-1/133 dKO embryos at E11.5 (A, B) and E12.5 (C, D). dKO embryos (B) show impaired blood circulation at E11.5 compared to WT embryos (A). No living dKO embyros (D) were found at E12.5. A WT embryo (C) at E12.5 is shown for comparison. Scale bars in (A, C) correspond to 2 mm.
(TIF)

Figure S4 Deletion of miR-1-2/133a-1 does not disturb expression of its host gene Mindbomb1 (*mib1*). RNA expression was analyzed by qRT-PCR using *mib1* specific primers with RNA isolated from embryonic hearts (E10.5) of WT and dKO animals (n = 4 WT/3 dKO). The data were normalized to HPRT expression. No significant change in the expression of *mib1* was detected. The oligonucleotides used for the qRT-PCR are directed to the exons flanking the intron containing the miR-1/133 coding region. The qRT-PCR indicates that the splicing of mib1 is not disturbed by the deletion of the miR-1-2/133a-1 coding region.
(TIF)

Figure S5 Loss of miR-1/133a leads to reduced proliferation rates in embryonic hearts. (A–D) Immunofluorescence analysis of EdU incorporation in hearts of wildtype (wt) and double cluster knockout (dKO) embryos at E9.5 (A, B) and E10.5 (C, D). A significant reduction of proliferating cells in dKO mutants is visible on sections through the heart. (E, F) Immunofluorescence analysis of proliferating pH3-positive myosin-expressing cardiomyo-cytes (green) in hearts of wildtype (wt) (E) and double cluster knockout (dKO) (F) embryos at E10.5. (G, H) Immunofluorescence analysis of increased sm-actin expression in hearts of double cluster knockout (dKO) (H) compared to wildtype (wt) embryos (G) at E10.5. The scale bar in (B) corresponds to 50 µm in (A, B, E–H); the scale bar in (D) corresponds to 50 µm in (C, D).
(TIF)

Figure S6 Gene ontology enrichment analysis of genes at least 1.5-fold up-regulated in miR-1/133a dKO compared to wt control hearts at E10.5. Hierarchically structured GO terms are ordered according to significance (p-value) of enrichment. GO terms that are not significantly enriched (p-value <0.05) are not shown. The most significant GO term of a hierarchy level is expanded. The GO terms “vasculogenesis”, “cardiomyocyte differentiation” and “muscle cell differentiation”, contained in the term “cell differentiation”, represent the most significant terms. Genes that are at least 1.5-fold up-regulated in dKO vs. control E10.5 hearts are clearly overrepresented in GO terms associated with smooth muscle gene differentiation compared to other GO terms.
(TIF)

Figure S7 Expression of cardiac and smooth muscle isoforms of myocardin in embryonic hearts of WT and miR-1/133a dKO embryos. RT-PCR analysis of expression of myocardin splice variants. Embryonic hearts of WT, homozygous miR-1-1/133a-2 or miR-1-2/133a-1 mutant mice (sKO) and homozygous miR-1-1/133a-2//miR-1-2/133a-1 dKO mice only express the cardiac splice variant of myocardin at E10.5. Expression of the smooth muscle specific isoform of myocardin in the bladder (b) and of the cardiac isoform in the adult heart of wild type mice are shown for comparison. Cardiac (238 bp) and smooth muscle specific isoforms (282 bp) were amplified using specific primer pairs. Gapdh served as a loading control.
(TIF)

Figure S8 Expression of Hand2 protein is not changed in the heart of dKO embryos at E10.5. Western blot analysis of 3 different pools of WT and dKO whole embryonic heart at E10.5 (representing 14 WT and 14 dKO samples) was performed to monitor Hand2 protein expression. The level of Hand2 was not changed in dKO compared to WT embryonic hearts.
(TIF)

Figure S9 Comparative expression analysis of genes in myocardin overexpressing NIH3T3 cells and in hearts of miR-1/133a dko embryonic hearts at E10.5. Affymetrix DNA microarray-based transcriptional analysis of myocardin-overexpressing NIH3T3 cells and miR-1/133a dKO mutant hearts. Fold changes relative to untransfected NIH3T3 cells or WT embryos are shown. Please note that several genes up-regulated in myocardin-overexpressing NIH3T3 cells were also upregulated in miR-1/133a dKO embryos. Kcnmb1 is a known primary transcriptional target of myocardin.
(TIF)

Figure S10 Transgenic expression of myocardin (tg) in embryonic hearts recapitulates transcriptional changes induced by deletion of miR-1/133a clusters (dKO) at E10.5. (A) Venn diagram of genes up-regulated at least 1.5-fold in dKO and myocardin overexpressing whole hearts. (B) DNA microarray-based transcriptional analysis of myocardin transgenic hearts at E10.5 and comparison to miR-1/133a mutants. Several genes that are significantly up-regulated in miR-1/133a dKO hearts are also up-regulated after transgenic expression of myocardin. Note that the miR-133a and miR-1 target genes Kcnmb1 and BMP-10 are not significantly up-regulated in myocardin transgenic embryonic hearts. miR-1/133a regulated genes that do not respond to myocardin overexpression are indicated.
(TIF)

Figure S11 BMP10 is a direct primary target of miR-1. Putative miR-1 WT and mutant binding sites located in the ORF of BMP-10 were cloned into the pmirGLO Dual-Luciferase Vector. miR-1 mediated suppression of luciferase activity via WT but not mutant miRNA binding sites located in the BMP-10 mRNA. Vectors were transfected into HEK293 cells. Firefly luciferase intensities were normalized to Renilla activities.
(TIF)

Table S1 Deletion of single miR-1/133a gene clusters does not lead to embryonic lethality. Outcome of matings of animals heterozygous for deletions of miR-1-1/133a-2 and miR-1-2/133a-1. The number of offspring showed the expected mendelian distribution of WT, heterozygous and homozygous animals. Mean survival rates after 200 days showed no differences between miR-1-1/133a-2 mutant mice (99%, n = 147), miR-1-2/133a-1 mutant mice (97%, n = 233) and WT littermates (97%, n = 340).
(DOCX)

Table S2 Deletion of both miR-1/133a clusters leads to embryonic lethality. Outcome of matings of compound heterozygous miR-1-1/133a-2 and miR-1-2/133a-1 mutant mice. No compound homozygous miR-1-1/133a-2//miR-1-2/133a-1 mutant mice were recovered at the newborn stage while the number of miR-1/133 dKO embryos matched the expected frequencies until E11.5. Only values for WT, double heterozygous and double homozygous (dKO) mutants are listed.
(DOCX)

Text S1 Supplemental experimental procedures.
(DOCX)

## References

[1] Moorman AF, Christoffels VM (2003) Cardiac chamber formation: development, genes, and evolution. Physiol Rev 83: 1223–1267. 14506305 10.1152/physrev.00006.2003

[2] Ruzicka DL, Schwartz RJ (1988) Sequential activation of alpha-actin genes during avian cardiogenesis: vascular smooth muscle alpha-actin gene transcripts mark the onset of cardiomyocyte differentiation. J Cell Biol 107: 2575–2586. 3204121 10.1083/jcb.107.6.2575 PMC2115638

[3] Li L, Miano JM, Cserjesi P, Olson EN (1996) SM22 alpha, a marker of adult smooth muscle, is expressed in multiple myogenic lineages during embryogenesis. Circ Res 78: 188–195. 8575061 10.1161/01.res.78.2.188

[4] Boettger T, Beetz N, Kostin S, Schneider J, Kruger M, et al (2009) Acquisition of the contractile phenotype by murine arterial smooth muscle cells depends on the Mir143/145 gene cluster. J Clin Invest 119: 2634–2647. 19690389 10.1172/JCI38864 PMC2735940

[5] Huang J, Elicker J, Bowens N, Liu X, Cheng L, et al (2012) Myocardin regulates BMP-10 expression and is required for heart development. J Clin Invest 122: 3678–3691. 22996691 10.1172/JCI63635 PMC3461917

[6] Boettger T, Braun T (2012) A new level of complexity: the role of microRNAs in cardiovascular development. Circ Res 110: 1000–1013. 22461364 10.1161/CIRCRESAHA.111.247742

[7] Wang C, Cao D, Wang Q, Wang DZ (2011) Synergistic activation of cardiac genes by myocardin and Tbx5. PLoS One 6: e24242. 21897873 10.1371/journal.pone.0024242 PMC3163680

[8] van Rooij E, Quiat D, Johnson BA, Sutherland LB, Qi X, et al (2009) A family of microRNAs encoded by myosin genes governs myosin expression and muscle performance. Dev Cell 17: 662–673. 19922871 10.1016/j.devcel.2009.10.013 PMC2796371

[9] Callis TE, Pandya K, Seok HY, Tang RH, Tatsuguchi M, et al (2009) MicroRNA-208a is a regulator of cardiac hypertrophy and conduction in mice. J Clin Invest 119: 2772–2786. 19726871 10.1172/JCI36154 PMC2735902

[10] Creemers EE, Sutherland LB, Oh J, Barbosa AC, Olson EN (2006) Coactivation of MEF2 by the SAP domain proteins myocardin and MASTR. Mol Cell 23: 83–96. 16818234 10.1016/j.molcel.2006.05.026

[11] Sweetman D, Rathjen T, Jefferson M, Wheeler G, Smith TG, et al (2006) FGF-4 signaling is involved in mir-206 expression in developing somites of chicken embryos. Dev Dyn 235: 2185–2191. 16804893 10.1002/dvdy.20881

[12] Chen JF, Mandel EM, Thomson JM, Wu Q, Callis TE, et al (2006) The role of microRNA-1 and microRNA-133 in skeletal muscle proliferation and differentiation. Nat Genet 38: 228–233. 16380711 10.1038/ng1725 PMC2538576

[13] Liu N, Williams AH, Kim Y, McAnally J, Bezprozvannaya S, et al (2007) An intragenic MEF2-dependent enhancer directs muscle-specific expression of microRNAs 1 and 133. Proc Natl Acad Sci U S A 104: 20844–20849. 18093911 10.1073/pnas.0710558105 PMC2409229

[14] Zhao Y, Samal E, Srivastava D (2005) Serum response factor regulates a muscle-specific microRNA that targets Hand2 during cardiogenesis. Nature 436: 214–220. 15951802 10.1038/nature03817

[15] Liu N, Bezprozvannaya S, Williams AH, Qi X, Richardson JA, et al (2008) microRNA-133a regulates cardiomyocyte proliferation and suppresses smooth muscle gene expression in the heart. Genes Dev 22: 3242–3254. 19015276 10.1101/gad.1738708 PMC2600761

[16] Zhao Y, Ransom JF, Li A, Vedantham V, von Drehle M, et al (2007) Dysregulation of cardiogenesis, cardiac conduction, and cell cycle in mice lacking miRNA-1-2. Cell 129: 303–317. 17397913 10.1016/j.cell.2007.03.030

[17] Ikeda S, He A, Kong SW, Lu J, Bejar R, et al (2009) MicroRNA-1 negatively regulates expression of the hypertrophy-associated calmodulin and Mef2a genes. Mol Cell Biol 29: 2193–2204. 19188439 10.1128/MCB.01222-08 PMC2663304

[18] Chen H, Shi S, Acosta L, Li W, Lu J, et al (2004) BMP-10 is essential for maintaining cardiac growth during murine cardiogenesis. Development 131: 2219–2231. 15073151 10.1242/dev.01094 PMC2628765

[19] Gassmann M, Casagranda F, Orioli D, Simon H, Lai C, et al (1995) Aberrant neural and cardiac development in mice lacking the ErbB4 neuregulin receptor. Nature 378: 390–394. 7477376 10.1038/378390a0

[20] Chen YH, Ishii M, Sucov HM, Maxson RE Jr (2008) Msx1 and Msx2 are required for endothelial-mesenchymal transformation of the atrioventricular cushions and patterning of the atrioventricular myocardium. BMC Dev Biol 8: 75. 18667074 10.1186/1471-213X-8-75 PMC2518925

[21] Guo C, Sun Y, Zhou B, Adam RM, Li X, et al (2011) A Tbx1-Six1/Eya1-Fgf8 genetic pathway controls mammalian cardiovascular and craniofacial morphogenesis. J Clin Invest 121: 1585–1595. 21364285 10.1172/JCI44630 PMC3069777

[22] Imamura M, Long X, Nanda V, Miano JM (2010) Expression and functional activity of four myocardin isoforms. Gene 464: 1–10. 20385216 10.1016/j.gene.2010.03.012

[23] Long X, Tharp DL, Georger MA, Slivano OJ, Lee MY, et al (2009) The smooth muscle cell-restricted KCNMB1 ion channel subunit is a direct transcriptional target of serum response factor and myocardin. J Biol Chem 284: 33671–33682. 19801679 10.1074/jbc.M109.050419 PMC2785209

[24] Pipes GC, Creemers EE, Olson EN (2006) The myocardin family of transcriptional coactivators: versatile regulators of cell growth, migration, and myogenesis. Genes Dev 20: 1545–1556. 16778073 10.1101/gad.1428006

[25] Huang J, Min Lu M, Cheng L, Yuan LJ, Zhu X, et al (2009) Myocardin is required for cardiomyocyte survival and maintenance of heart function. Proc Natl Acad Sci U S A 106: 18734–18739. 19850880 10.1073/pnas.0910749106 PMC2773995

[26] Xing W, Zhang TC, Cao D, Wang Z, Antos CL, et al (2006) Myocardin induces cardiomyocyte hypertrophy. Circ Res 98: 1089–1097. 16556869 10.1161/01.RES.0000218781.23144.3e

[27] Wang Z, Wang DZ, Pipes GC, Olson EN (2003) Myocardin is a master regulator of smooth muscle gene expression. Proc Natl Acad Sci U S A 100: 7129–7134. 12756293 10.1073/pnas.1232341100 PMC165841

[28] Long X, Bell RD, Gerthoffer WT, Zlokovic BV, Miano JM (2008) Myocardin is sufficient for a smooth muscle-like contractile phenotype. Arterioscler Thromb Vasc Biol 28: 1505–1510. 18451334 10.1161/ATVBAHA.108.166066 PMC2574857

[29] Boogerd CJ, Moorman AF, Barnett P (2010) Expression of muscle segment homeobox genes in the developing myocardium. Anat Rec (Hoboken) 293: 998–1001. 20225205 10.1002/ar.21112

[30] Consortium EP, Dunham I, Kundaje A, Aldred SF, Collins PJ, et al (2012) An integrated encyclopedia of DNA elements in the human genome. Nature 489: 57–74. 22955616 10.1038/nature11247 PMC3439153

[31] Meunier J, Lemoine F, Soumillon M, Liechti A, Weier M, et al (2013) Birth and expression evolution of mammalian microRNA genes. Genome Res 23: 34–45. 23034410 10.1101/gr.140269.112 PMC3530682

[32] Berndt JD, Aoyagi A, Yang P, Anastas JN, Tang L, et al (2011) Mindbomb 1, an E3 ubiquitin ligase, forms a complex with RYK to activate Wnt/beta-catenin signaling. J Cell Biol 194: 737–750. 21875946 10.1083/jcb.201107021 PMC3171123

[33] Kang K, Lee D, Hong S, Park SG, Song MR (2012) The E3 ligase mindbomb-1 (Mib1) modulates Delta-Notch signaling to control neurogenesis and gliogenesis in the developing spinal cord. J Biol Chem 288: 2580–2592. 23223237 10.1074/jbc.M112.398263 PMC3554925

[34] Li S, Wang DZ, Wang Z, Richardson JA, Olson EN (2003) The serum response factor coactivator myocardin is required for vascular smooth muscle development. Proc Natl Acad Sci U S A 100: 9366–9370. 12867591 10.1073/pnas.1233635100 PMC170924

[35] Hoofnagle MH, Neppl RL, Berzin EL, Teg Pipes GC, Olson EN, et al (2011) Myocardin is differentially required for the development of smooth muscle cells and cardiomyocytes. Am J Physiol Heart Circ Physiol 300: H1707–1721. 21357509 10.1152/ajpheart.01192.2010 PMC3094091

[36] Torrado M, Lopez E, Centeno A, Medrano C, Castro-Beiras A, et al (2003) Myocardin mRNA is augmented in the failing myocardium: expression profiling in the porcine model and human dilated cardiomyopathy. J Mol Med (Berl) 81: 566–577. 12920479 10.1007/s00109-003-0470-7

[37] Larson AC, White RD, Laub G, McVeigh ER, Li D, et al (2004) Self-gated cardiac cine MRI. Magnetic resonance in medicine : official journal of the Society of Magnetic Resonance in Medicine/Society of Magnetic Resonance in Medicine 51: 93–102. 10.1002/mrm.10664 PMC2396326 14705049

