# Supplementary figures and images for: miR-1/133a Clusters Cooperatively Specify the Cardiomyogenic Lineage by Adjustment of Myocardin Levels during Embryonic Heart Development
Source: PLoS Genet. 2013 Sep 19;9(9):e1003793. doi: 10.1371/journal.pgen.1003793 (PMC3777988; doi:10.1371/journal.pgen.1003793)

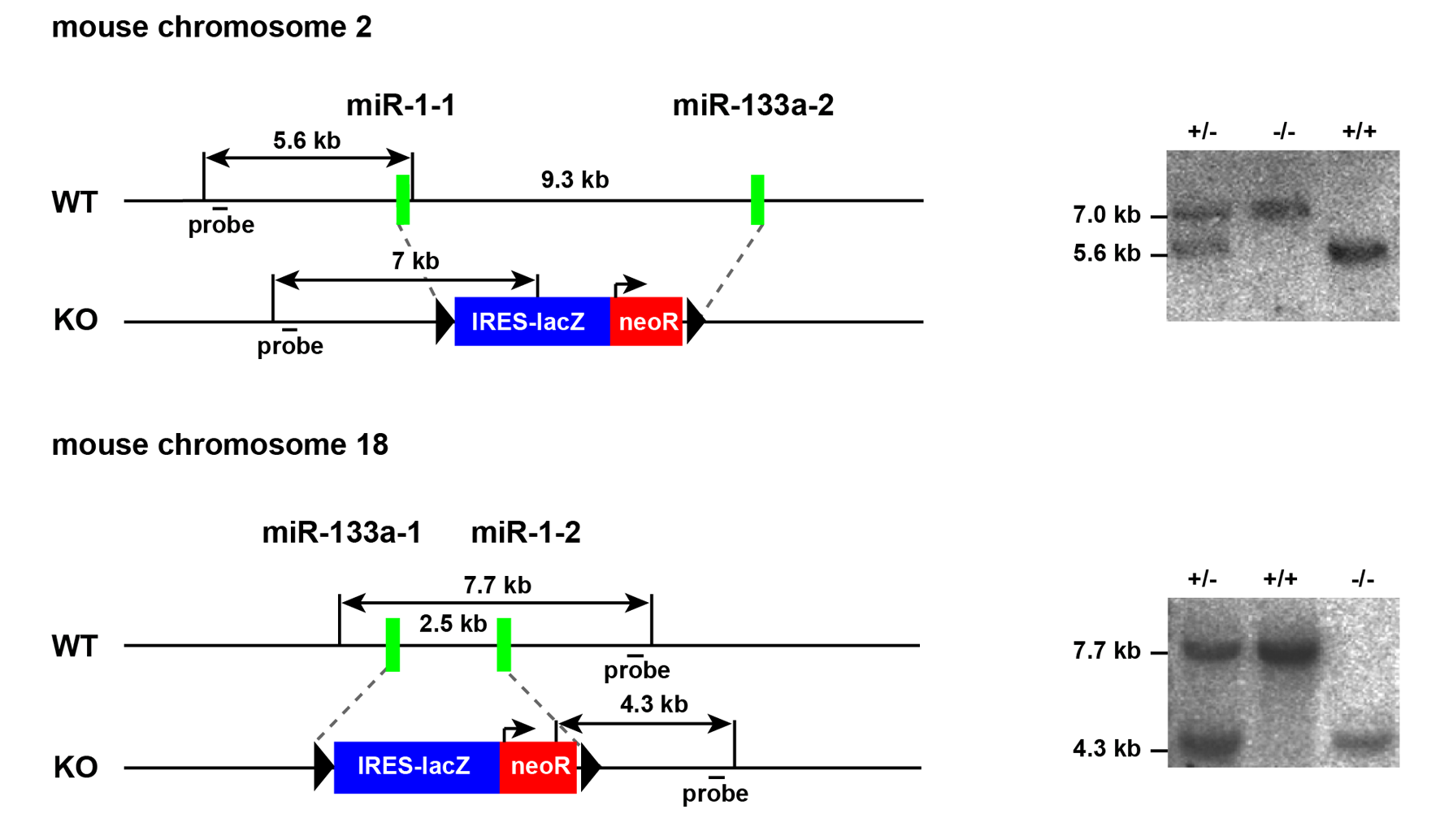

Supplement: Figure S1 — Deletion of miR-1/133a coding clusters on mouse chromosome 2 and chromosome 18. The genomic regions coding for miR-1-1/133a-2 on mouse chromosome 2 and miR-1-2/133a-1 on mouse chromosome 18 were replaced with loxP-flanked IRES-LacZ/neoR cassettes. Recombination of the genomic locus was analyzed using probes located at genomic regions outside of the respective targeting vectors. Localization of the Southern blot probes is indicated. Both mouse lines were bred to generate homozygous offspring. (TIF) [file pgen.1003793.s001.tif]

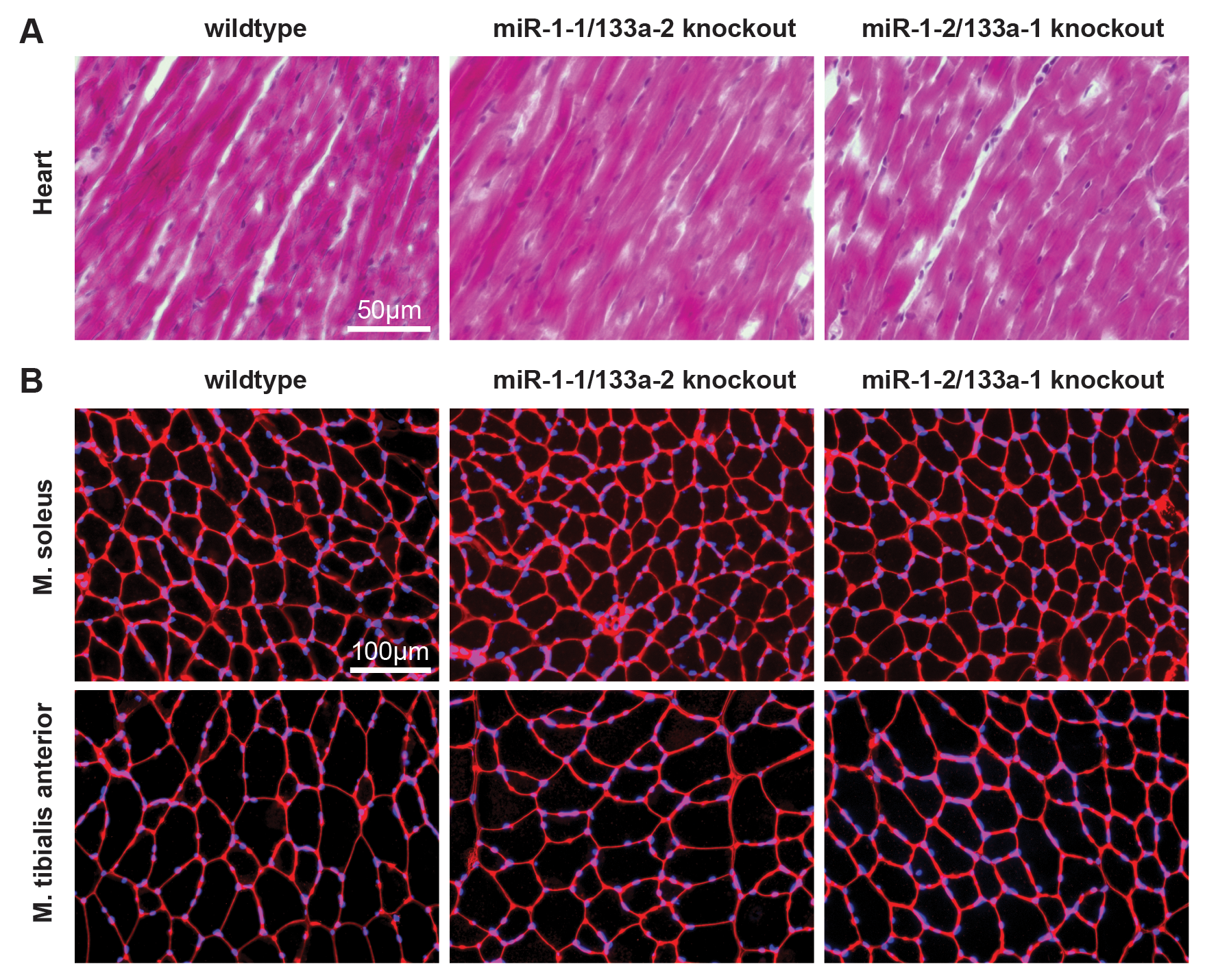

Supplement: Figure S2 — Deletion of single miR-1/133a genomic clusters does not lead to gross morphological alterations in heart and skeletal muscle. (A) HE stained transverse sections of the ventricle reveal no histological abnormalities. The scale bar in (A) corresponds to 50 µm. (B) Immunofluorescence analysis of skeletal muscles of miR-1-1/mirR-133a-2 and miR-1-2/mirR-133a-1 homozygous mutant mice. No increase of cellularity, centrally located nuclei indicating regeneration or changes in the diameter of myotubes are visible on cross sections stained with Triticum vulagaris lectin and DAPI. The scale bar in (B) corresponds to 100 µm. (TIF) [file pgen.1003793.s002.tif]

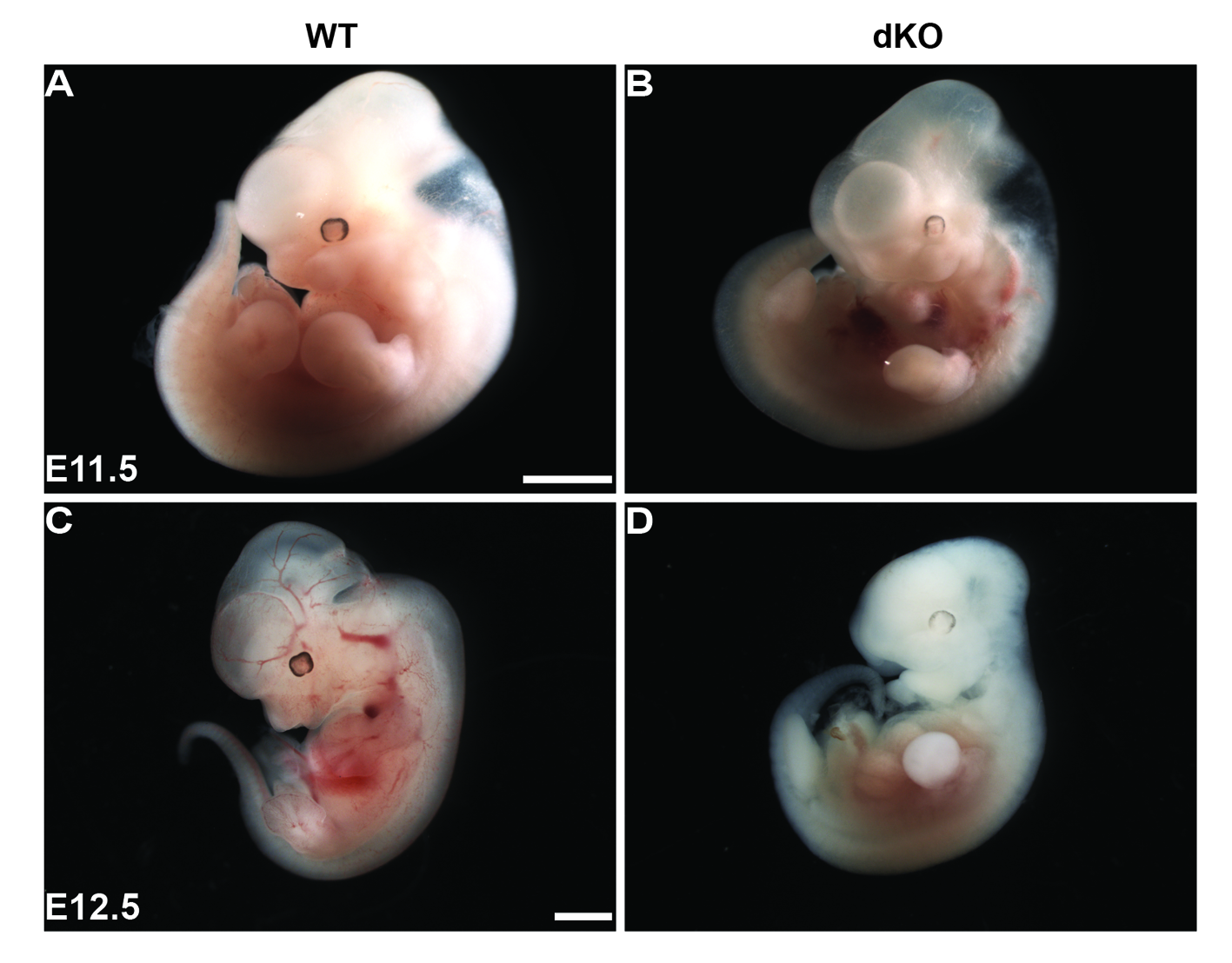

Supplement: Figure S3 — Deletion of both miR-1/133a clusters leads to arrest of heart development and embryonic lethality. (A–D) Macroscopic views of isolated wild type (WT) and miR-1/133 dKO embryos at E11.5 (A, B) and E12.5 (C, D). dKO embryos (B) show impaired blood circulation at E11.5 compared to WT embryos (A). No living dKO embyros (D) were found at E12.5. A WT embryo (C) at E12.5 is shown for comparison. Scale bars in (A, C) correspond to 2 mm. (TIF) [file pgen.1003793.s003.tif]

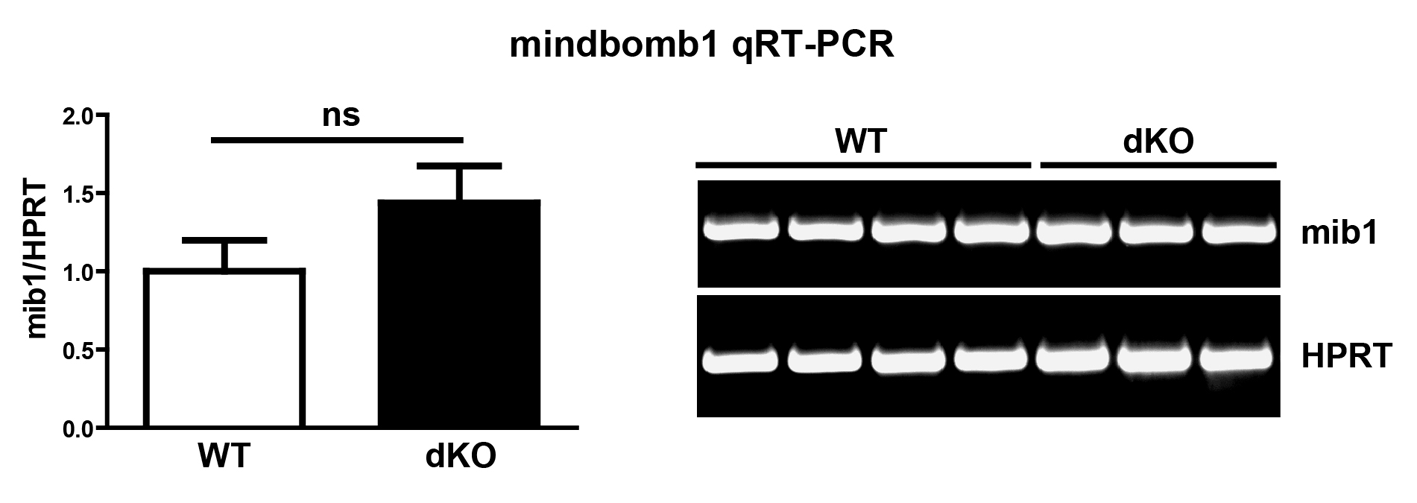

Supplement: Figure S4 — Deletion of miR-1-2/133a-1 does not disturb expression of its host gene Mindbomb1 (mib1). RNA expression was analyzed by qRT-PCR using mib1 specific primers with RNA isolated from embryonic hearts (E10.5) of WT and dKO animals (n = 4 WT/3 dKO). The data were normalized to HPRT expression. No significant change in the expression of mib1 was detected. The oligonucleotides used for the qRT-PCR are directed to the exons flanking the intron containing the miR-1/133 coding region. The qRT-PCR indicates that the splicing of mib1 is not disturbed by the deletion of the miR-1-2/133a-1 coding region. (TIF) [file pgen.1003793.s004.tif]

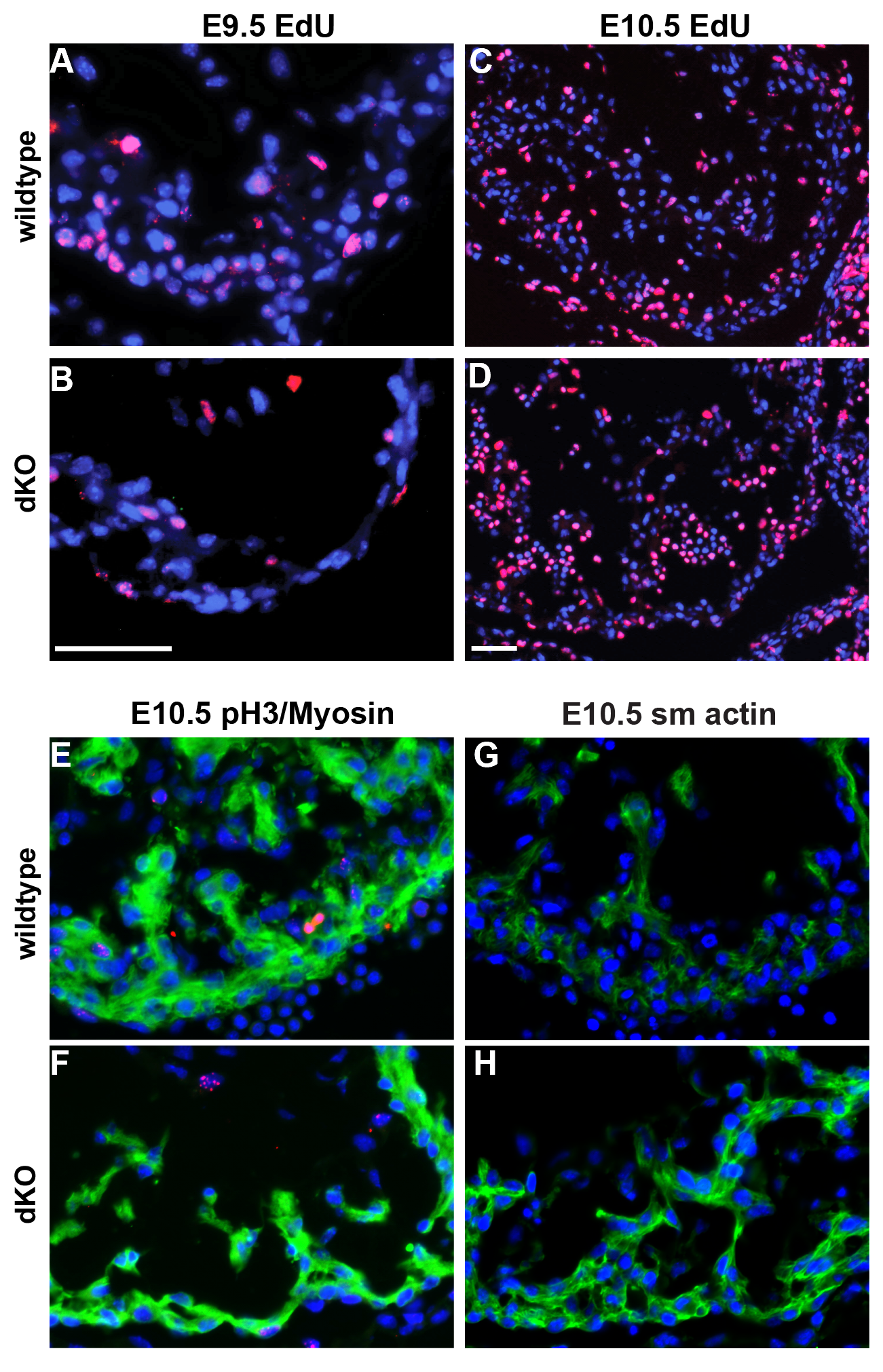

Supplement: Figure S5 — Loss of miR-1/133a leads to reduced proliferation rates in embryonic hearts. (A–D) Immunofluorescence analysis of EdU incorporation in hearts of wildtype (wt) and double cluster knockout (dKO) embryos at E9.5 (A, B) and E10.5 (C, D). A significant reduction of proliferating cells in dKO mutants is visible on sections through the heart. (E, F) Immunofluorescence analysis of proliferating pH3-positive myosin-expressing cardiomyo-cytes (green) in hearts of wildtype (wt) (E) and double cluster knockout (dKO) (F) embryos at E10.5. (G, H) Immunofluorescence analysis of increased sm-actin expression in hearts of double cluster knockout (dKO) (H) compared to wildtype (wt) embryos (G) at E10.5. The scale bar in (B) corresponds to 50 µm in (A, B, E–H); the scale bar in (D) corresponds to 50 µm in (C, D). (TIF) [file pgen.1003793.s005.tif]

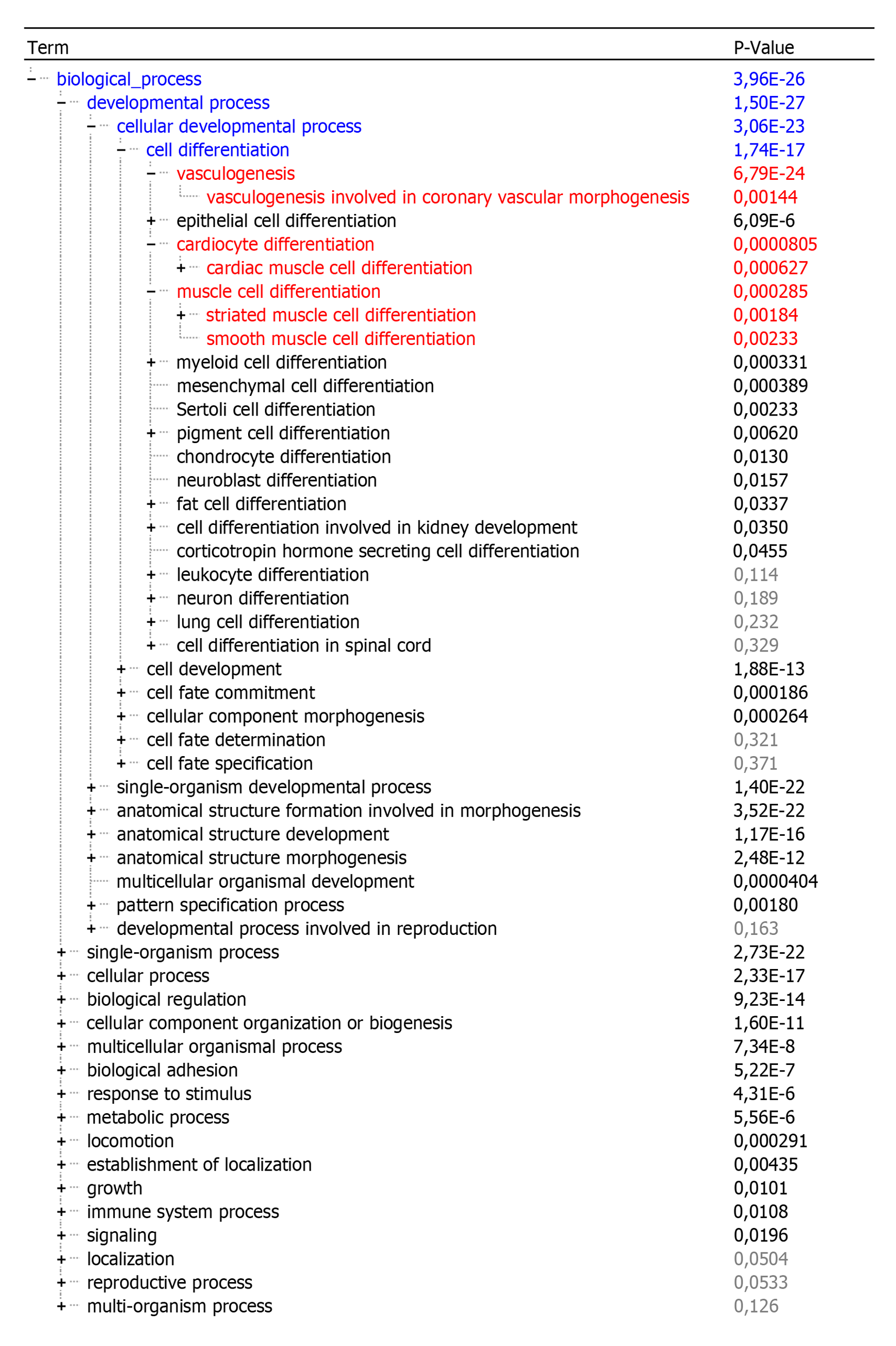

Supplement: Figure S6 — Gene ontology enrichment analysis of genes at least 1.5-fold up-regulated in miR-1/133a dKO compared to wt control hearts at E10.5. Hierarchically structured GO terms are ordered according to significance (p-value) of enrichment. GO terms that are not significantly enriched (p-value <0.05) are not shown. The most significant GO term of a hierarchy level is expanded. The GO terms “vasculogenesis”, “cardiomyocyte differentiation” and “muscle cell differentiation”, contained in the term “cell differentiation”, represent the most significant terms. Genes that are at least 1.5-fold up-regulated in dKO vs. control E10.5 hearts are clearly overrepresented in GO terms associated with smooth muscle gene differentiation compared to other GO terms. (TIF) [file pgen.1003793.s006.tif]

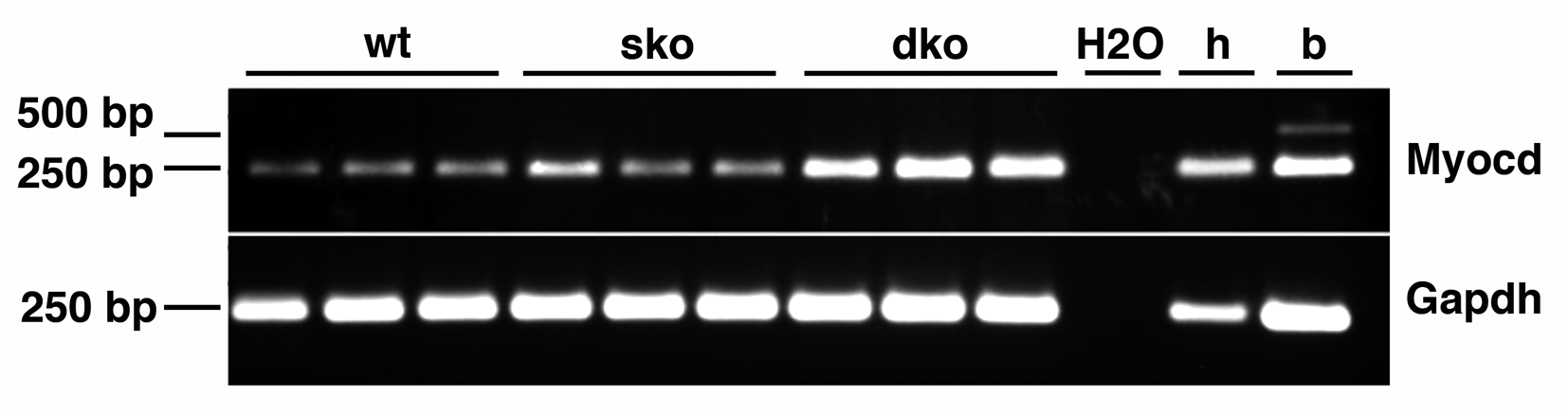

Supplement: Figure S7 — Expression of cardiac and smooth muscle isoforms of myocardin in embryonic hearts of WT and miR-1/133a dKO embryos. RT-PCR analysis of expression of myocardin splice variants. Embryonic hearts of WT, homozygous miR-1-1/133a-2 or miR-1-2/133a-1 mutant mice (sKO) and homozygous miR-1-1/133a-2//miR-1-2/133a-1 dKO mice only express the cardiac splice variant of myocardin at E10.5. Expression of the smooth muscle specific isoform of myocardin in the bladder (b) and of the cardiac isoform in the adult heart of wild type mice are shown for comparison. Cardiac (238 bp) and smooth muscle specific isoforms (282 bp) were amplified using specific primer pairs. Gapdh served as a loading control. (TIF) [file pgen.1003793.s007.tif]

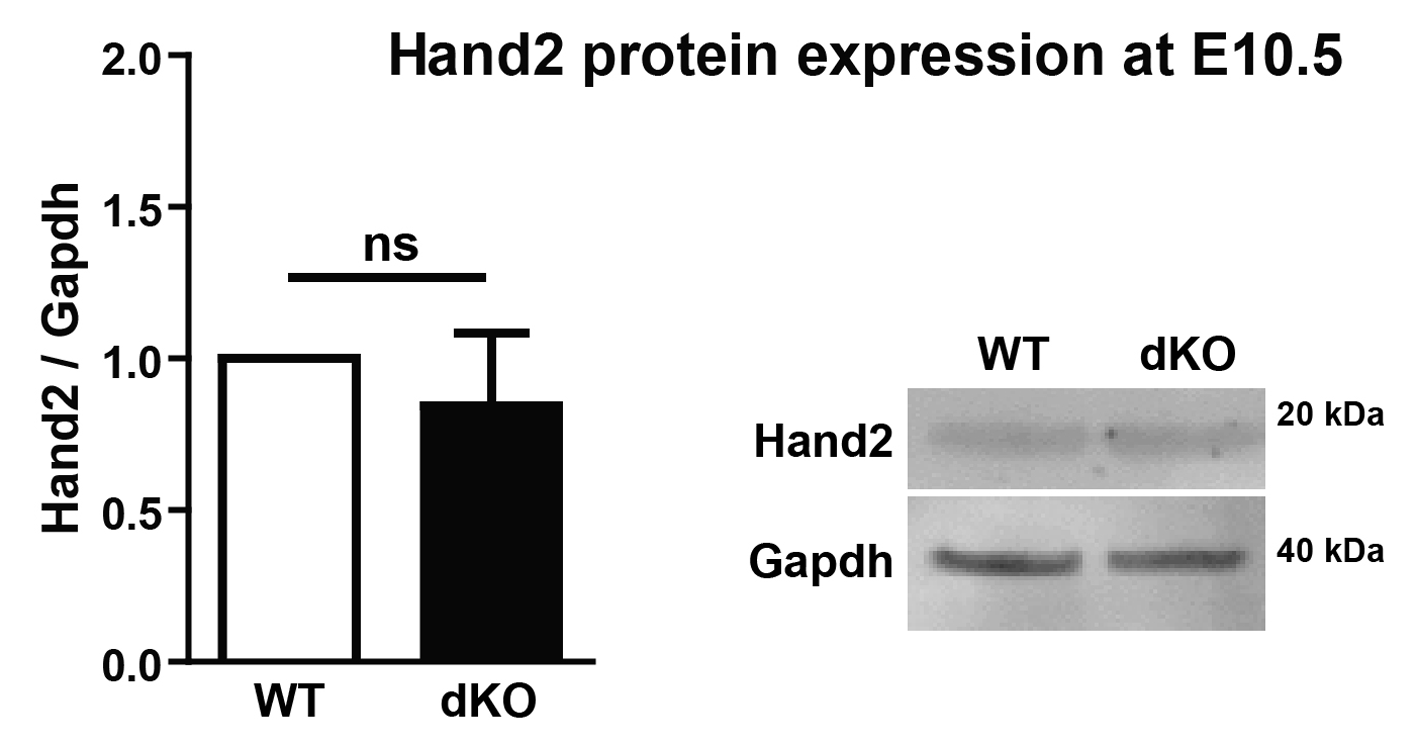

Supplement: Figure S8 — Expression of Hand2 protein is not changed in the heart of dKO embryos at E10.5. Western blot analysis of 3 different pools of WT and dKO whole embryonic heart at E10.5 (representing 14 WT and 14 dKO samples) was performed to monitor Hand2 protein expression. The level of Hand2 was not changed in dKO compared to WT embryonic hearts. (TIF) [file pgen.1003793.s008.tif]

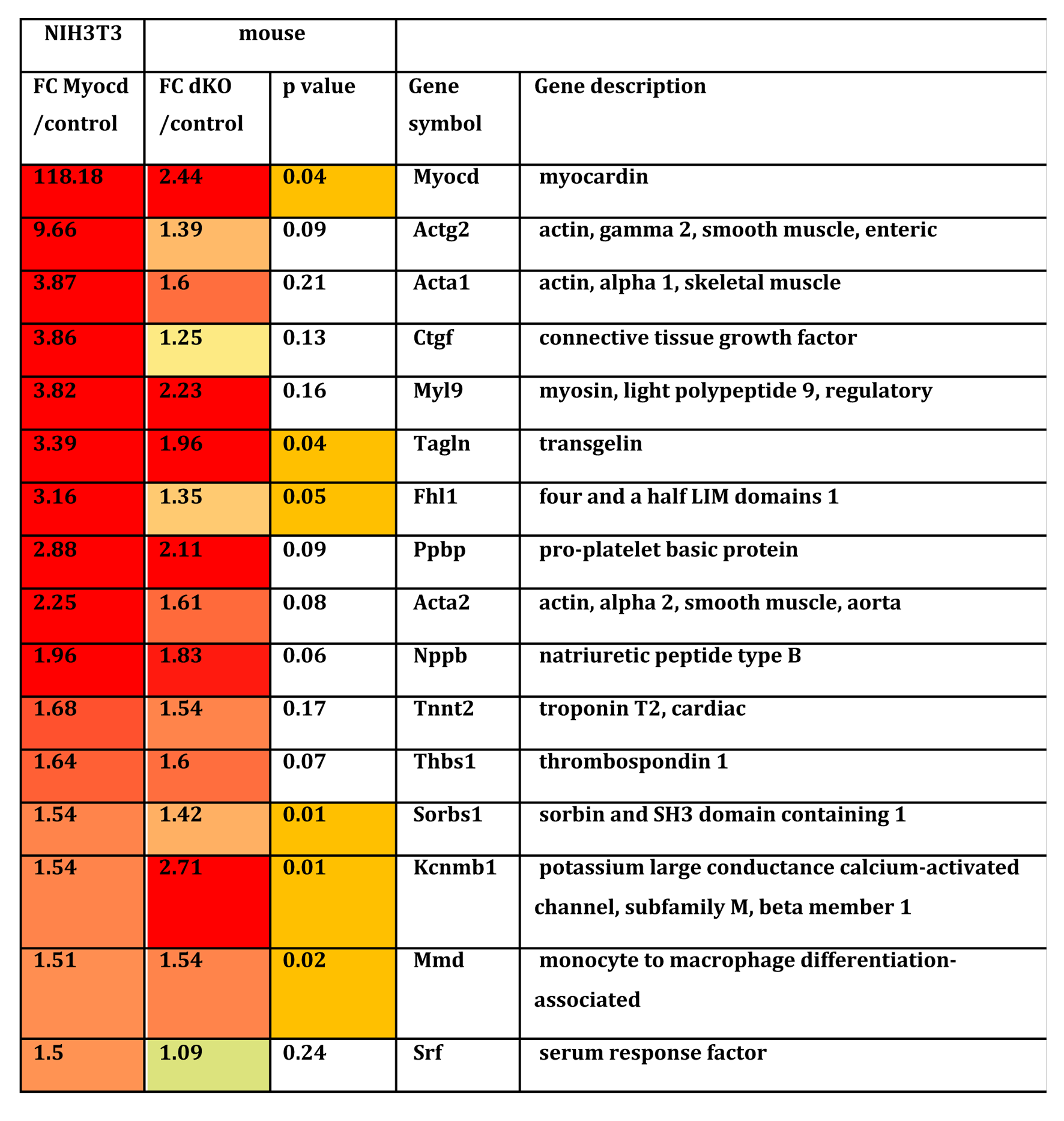

Supplement: Figure S9 — Comparative expression analysis of genes in myocardin overexpressing NIH3T3 cells and in hearts of miR-1/133a dko embryonic hearts at E10.5. Affymetrix DNA microarray-based transcriptional analysis of myocardin-overexpressing NIH3T3 cells and miR-1/133a dKO mutant hearts. Fold changes relative to untransfected NIH3T3 cells or WT embryos are shown. Please note that several genes up-regulated in myocardin-overexpressing NIH3T3 cells were also upregulated in miR-1/133a dKO embryos. Kcnmb1 is a known primary transcriptional target of myocardin. (TIF) [file pgen.1003793.s009.tif]

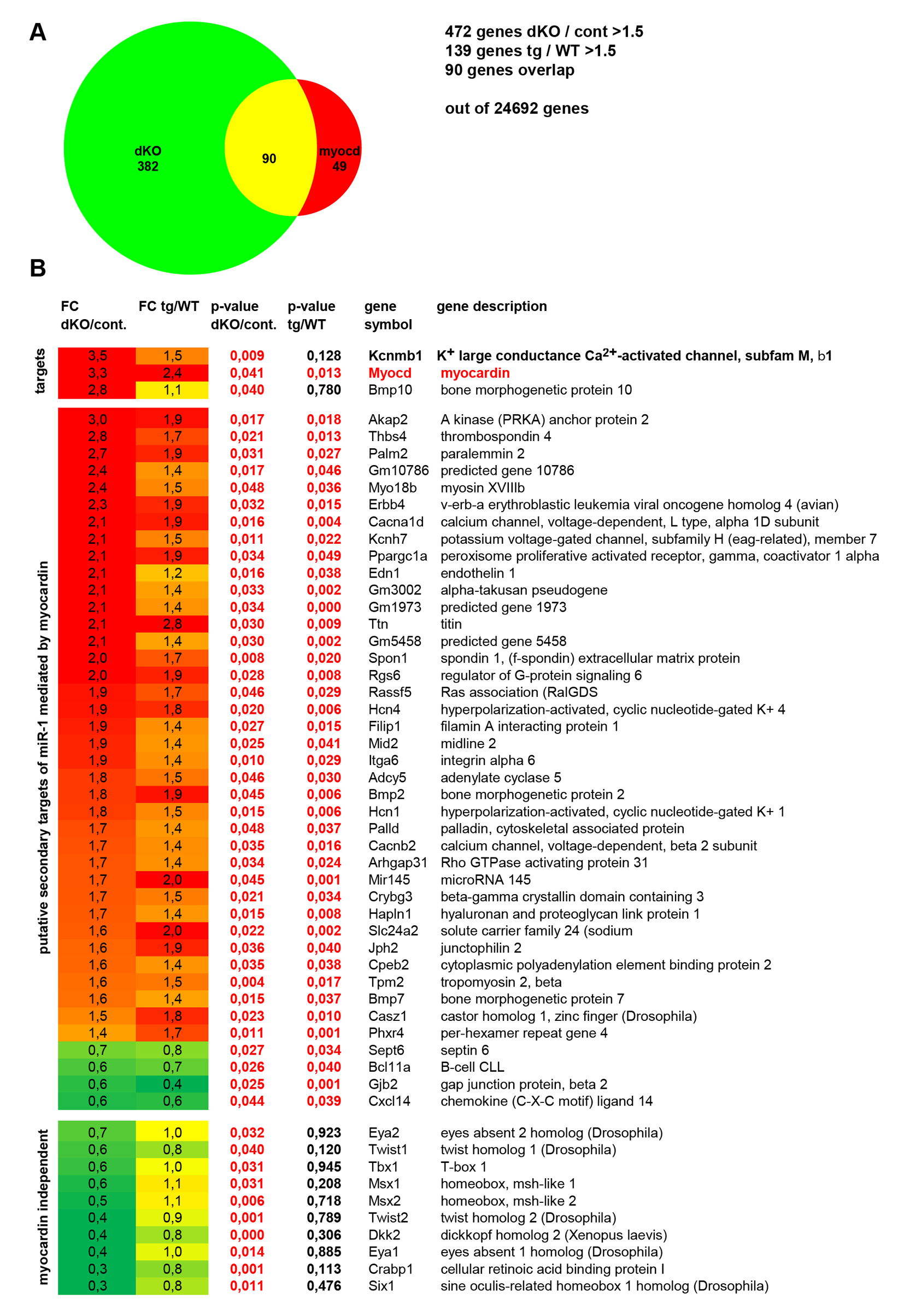

Supplement: Figure S10 — Transgenic expression of myocardin (tg) in embryonic hearts recapitulates transcriptional changes induced by deletion of miR-1/133a clusters (dKO) at E10.5. (A) Venn diagram of genes up-regulated at least 1.5-fold in dKO and myocardin overexpressing whole hearts. (B) DNA microarray-based transcriptional analysis of myocardin transgenic hearts at E10.5 and comparison to miR-1/133a mutants. Several genes that are significantly up-regulated in miR-1/133a dKO hearts are also up-regulated after transgenic expression of myocardin. Note that the miR-133a and miR-1 target genes Kcnmb1 and BMP-10 are not significantly up-regulated in myocardin transgenic embryonic hearts. miR-1/133a regulated genes that do not respond to myocardin overexpression are indicated. (TIF) [file pgen.1003793.s010.tif]

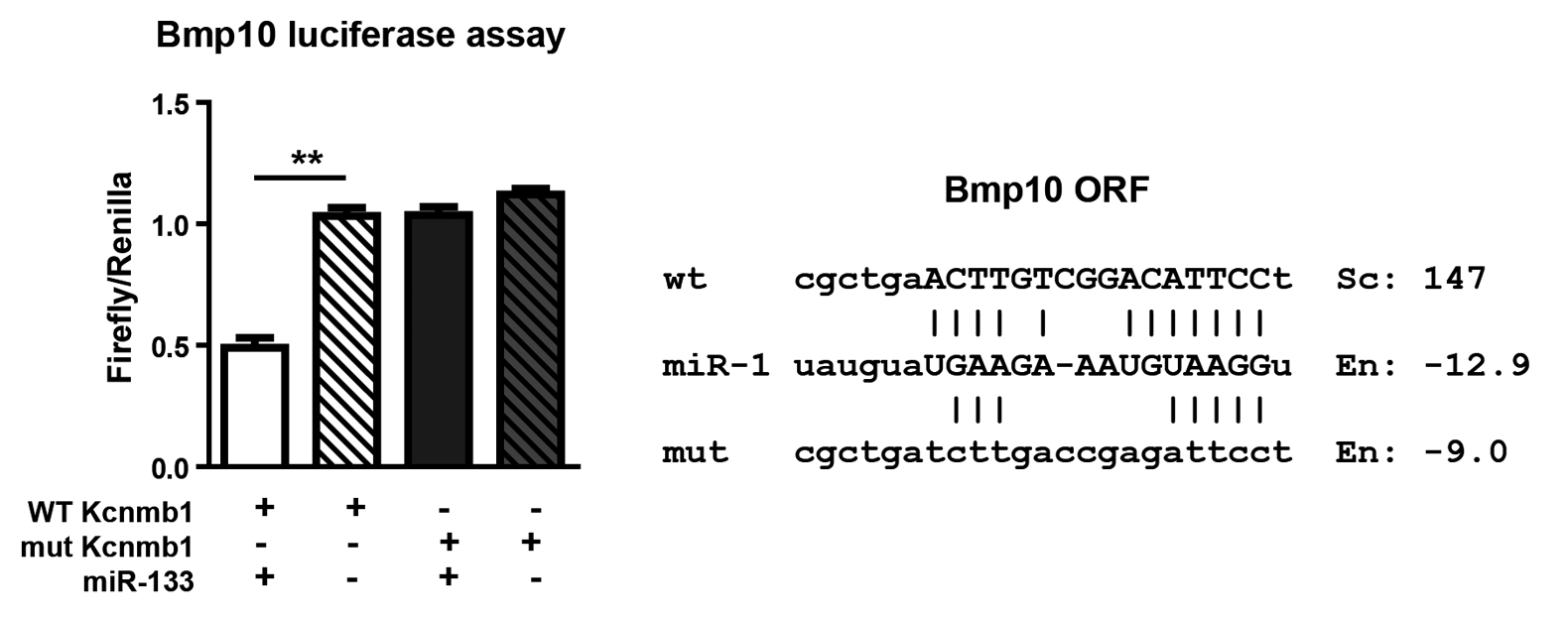

Supplement: Figure S11 — BMP10 is a direct primary target of miR-1. Putative miR-1 WT and mutant binding sites located in the ORF of BMP-10 were cloned into the pmirGLO Dual-Luciferase Vector. miR-1 mediated suppression of luciferase activity via WT but not mutant miRNA binding sites located in the BMP-10 mRNA. Vectors were transfected into HEK293 cells. Firefly luciferase intensities were normalized to Renilla activities. (TIF) [file pgen.1003793.s011.tif]
