# Supplementary material for: miR-1/133a Clusters Cooperatively Specify the Cardiomyogenic Lineage by Adjustment of Myocardin Levels during Embryonic Heart Development
Source: PLoS Genet. 2013 Sep 19;9(9):e1003793. doi: 10.1371/journal.pgen.1003793 (PMC3777988; doi:10.1371/journal.pgen.1003793)
Supplement: Table S1 — Deletion of single miR-1/133a gene clusters does not lead to embryonic lethality. Outcome of matings of animals heterozygous for deletions of miR-1-1/133a-2 and miR-1-2/133a-1. The number of offspring showed the expected mendelian distribution of WT, heterozygous and homozygous animals. Mean survival rates after 200 days showed no differences between miR-1-1/133a-2 mutant mice (99%, n = 147), miR-1-2/133a-1 mutant mice (97%, n = 233) and WT littermates (97%, n = 340). (DOCX) [file pgen.1003793.s012.docx]

|  | **miR-1-1/133a-2**  **chromosome 18** | | **miR-1-2/133a-1**  **chromosome 2** | |
| --- | --- | --- | --- | --- |
| **+/+** | **46** | **24.9%** | **50** | **22.0%** |
| **+/-** | **96** | **51.9%** | **111** | **48.9%** |
| **-/-** | **43** | **23.2%** | **66** | **29.1%** |
| **total** | **185** | **100.0%** | **227** | **100.0%** |
