# Supplementary material for: miR-1/133a Clusters Cooperatively Specify the Cardiomyogenic Lineage by Adjustment of Myocardin Levels during Embryonic Heart Development
Source: PLoS Genet. 2013 Sep 19;9(9):e1003793. doi: 10.1371/journal.pgen.1003793 (PMC3777988; doi:10.1371/journal.pgen.1003793)
Supplement: Table S2 — Deletion of both miR-1/133a clusters leads to embryonic lethality. Outcome of matings of compound heterozygous miR-1-1/133a-2 and miR-1-2/133a-1 mutant mice. No compound homozygous miR-1-1/133a-2//miR-1-2/133a-1 mutant mice were recovered at the newborn stage while the number of miR-1/133 dKO embryos matched the expected frequencies until E11.5. Only values for WT, double heterozygous and double homozygous (dKO) mutants are listed. (DOCX) [file pgen.1003793.s013.docx]

| **Age** | **Total mice** |  | **WT** | **hetero/hetero** | **dKO** |
| --- | --- | --- | --- | --- | --- |
| **Newborn** | 116 | observed | 7 | 24 | 0 |
|  |  | expected | 4 | 16 | 4 |
| **E9.5 – 11.5** | 64 | observed | 7 | 14 | 6 |
|  |  | expected | 4 | 16 | 4 |
