# Supplementary material for: miR-1/133a Clusters Cooperatively Specify the Cardiomyogenic Lineage by Adjustment of Myocardin Levels during Embryonic Heart Development
Source: PLoS Genet. 2013 Sep 19;9(9):e1003793. doi: 10.1371/journal.pgen.1003793 (PMC3777988; doi:10.1371/journal.pgen.1003793)
Supplement: Text S1 — Supplemental experimental procedures. (DOCX) [file pgen.1003793.s014.docx]

**Supplemental experimental procedures**

**Mib1 qRT-PCR**

Abundance of mindbomb1 in the E10.5 embryonic heart of WT and dKO embryos was determined by qRT-PCR using the oligonucleotides for mib1 (gtcat­cccag­tctcc­agga­ttctg­aa, ggacc­aaaag­cctaa­caatc­tgggt). The RT-PCR was performed using a FastStart Universal SYBR-Green Mastermix (Roche) to allow quantification. Data were normalized to HPRT detection (gctgg­tgaaa­agga­cct­ct, caca­ggac­taga­acacc­tgc). Abundance of mib1 was calculated using the ddCt method. Unpaired Student´s t-test was performed. The identity of the PCR products was analyzed on an Agarose gel to exclude potential disturbance of mib1-splicing.

**Lectin staining of muscle sections**

Adult muscle samples were dissected, washed in PBS, and flash-frozen in liquid nitrogen cooled propane/methybutan (1:2). Muscles were embedded in Tissue Tek, cryosectioned and mounted on Superfrost slides. Sections were fixed in 4% PFA, and washed with PBS before staining with DAPI and TRITC conjugated Triticum vulgaris - Lectin (Sigma#L5266; 1:200). Sections were embedded in Fluoromount. Pictures were taken using a Z1 Axioimager (Zeiss).

### BMP10 Luciferase reporter assays

WT and mutated miRNA binding sites were directionally cloned in quadruplicate into the NheI and XhoI sites of the pmirGLO Dual-Luciferase Vector (E13330, Promega) using synthetic oligonucleotides.

(BMP10 miR1 binding site:

CTAGCACGCTGAACTTGTCGGACATTCCTGTGAGACGCT AACTTGTCGGACATTCCTGTGAGACGCTGAACTTGTCGGACATTC TGTGAGAC CTGAACTTGTCGGACATTCC TGC.

BMP10 mutant miR-1 binding site:

CTAGCACGCTGATCTTGACCGAGATTCCTGTGAGACGCTGATCTTGACCGAGATTCCTGTGAGACGCTGATCTTGACCGAGATTCCTGTGAGACGCTGATCTTGACCGAGATTCCTGC.

70%-confluent HEK293 cells were transfected with 50 ng of the respective plasmid with 50 pmol of miRIDIAN microRNA mimic miR-1 (Thermo) using Lipofectamine 2000 (Invitrogen). Each transfection was done in triplicate. Cells were lysed 24 h after transfection. Firefly luciferase and renilla activities were determined using the Dual-Luciferase Reporter assay (Promega) and the Mithras LB940plate reader (Berthold). Firefly luciferase intensities were normalized to Renilla activities.
